# Variants encoding a restricted carboxy-terminal domain of *SLC12A2* cause hereditary hearing loss in humans

**DOI:** 10.1371/journal.pgen.1008643

**Published:** 2020-04-15

**Authors:** Hideki Mutai, Koichiro Wasano, Yukihide Momozawa, Yoichiro Kamatani, Fuyuki Miya, Sawako Masuda, Noriko Morimoto, Kiyomitsu Nara, Satoe Takahashi, Tatsuhiko Tsunoda, Kazuaki Homma, Michiaki Kubo, Tatsuo Matsunaga

**Affiliations:** 1 Division of Hearing and Balance Research, National Institute of Sensory Organs, National Hospital Organization Tokyo Medical Center, Meguro, Tokyo, Japan; 2 Department of Otolaryngology-Head and Neck Surgery, Northwestern University Feinberg School of Medicine, Chicago, Illinois, United States of America; 3 Laboratory for Genotyping Development, RIKEN Center for Integrative Medical Sciences, Yokohama, Kanagawa, Japan; 4 Laboratory for Statistical Analysis, RIKEN Center for Integrative Medical Sciences, Yokohama, Kanagawa, Japan; 5 Kyoto-McGill International Collaborative School in Genomic Medicine, Graduate School of Medicine, Kyoto University, Yoshidakonoecho, Kyoto, Japan; 6 Laboratory for Medical Science Mathematics, RIKEN Center for Integrative Medical Sciences, Yokohama, Kanagawa, Japan; 7 Department of Medical Science Mathematics, Medical Research Institute, Tokyo Medical and Dental University, Bunkyo, Tokyo, Japan; 8 Department of Otorhinolaryngology, National Hospital Organization Mie National Hospital, Tsu, Mie, Japan; 9 Department of Otorhinolaryngology, National Center for Child Health and Development, Setagaya, Tokyo, Japan; 10 Laboratory for Medical Science Mathematics, Department of Biological Sciences, Graduate School of Science, The University of Tokyo, Bunkyo, Tokyo, Japan; 11 The Hugh Knowles Center for Clinical and Basic Science in Hearing and Its Disorders, Northwestern University, Evanston, Illinois, United States of America; 12 RIKEN Center for Integrative Medical Sciences, Yokohama, Kanagawa, Japan; 13 Medical Genetics Center, National Hospital Organization Tokyo Medical Center, Meguro, Tokyo, Japan; University of Pennsylvania, UNITED STATES

## Abstract

Hereditary hearing loss is challenging to diagnose because of the heterogeneity of the causative genes. Further, some genes involved in hereditary hearing loss have yet to be identified. Using whole-exome analysis of three families with congenital, severe-to-profound hearing loss, we identified a missense variant of *SLC12A2* in five affected members of one family showing a dominant inheritance mode, along with *de novo* splice-site and missense variants of *SLC12A2* in two sporadic cases, as promising candidates associated with hearing loss. Furthermore, we detected another *de novo* missense variant of *SLC12A2* in a sporadic case. *SLC12A2* encodes Na^+^, K^+^, 2Cl^−^ cotransporter (NKCC) 1 and plays critical roles in the homeostasis of K^+^-enriched endolymph. *Slc12a2*-deficient mice have congenital, profound deafness; however, no human variant of *SLC12A2* has been reported as associated with hearing loss. All identified *SLC12A2* variants mapped to exon 21 or its 3’-splice site. *In vitro* analysis indicated that the splice-site variant generates an exon 21-skipped *SLC12A2* mRNA transcript expressed at much lower levels than the exon 21-included transcript in the cochlea, suggesting a tissue-specific role for the exon 21-encoded region in the carboy-terminal domain. *In vitro* functional analysis demonstrated that Cl^−^ influx was significantly decreased in all SLC12A2 variants studied. Immunohistochemistry revealed that SLC12A2 is located on the plasma membrane of several types of cells in the cochlea, including the strial marginal cells, which are critical for endolymph homeostasis. Overall, this study suggests that variants affecting exon 21 of the *SLC12A2* transcript are responsible for hereditary hearing loss in humans.

## Author summary

Sounds are perceived by auditory sensory cells, owing to tissues surrounding them, including the cochlear lateral wall. Part of the cochlear lateral wall, the stria vascularis, is critical for production and maintenance of inner-ear fluid with a high potassium concentration, and for generating the positive voltage in the inner ear, important for sound perception, by stimulating secretion of potassium from marginal cells. The gene *SLC12A2* encodes a protein involved in sodium, potassium, and chloride transport essential for proper function of specific cells in the stria vascularis; however, human variants of *SLC12A2* have not previously been associated with hearing loss. By comprehensive genetic analysis of protein-coding sequences, we identified four candidate changes in *SLC12A2* in four families with congenital, severe-to-profound hearing loss. Intriguingly, all four genetic variants were either within or at the 3’-splice site of the exon 21 which encodes a part of the carboxy terminal intracellular domain of SLC12A2. Experiments in cultured cells showed that skipping or mutation of exon 21 significantly decreased chloride influx mediated by the SLC12A2 protein. Overall, our results strongly indicate that mutations influencing exon 21 of *SLC12A2* represent a novel mechanism underlying deafness in humans.

## Introduction

Sensorineural hearing loss is one of the most common sensory disorders in humans, and its onset can be influenced by multiple environmental and genetic factors. Approximately 1 in 500 to 1,000 newborns has congenital sensorineural hearing loss [1], and more than 100 genes have been reported as associated with nonsyndromic hearing loss to date [2].

Hair bundles of auditory sensory cells (hair cells) in the mammalian cochlea are bathed in K^+^-rich endolymph at the apical membrane. Proper depolarization of hair cells is achieved via the influx of K^+^ from the endolymph through a mechanotransduction channel located on the hair bundles in response to auditory stimuli. Production and maintenance of endolymph with a high K^+^ concentration (> 150 mM) and endocochlear potential (> +80 mV) is primarily accomplished by the secretion of K^+^ from the marginal cells of the stria vascularis in the cochlear lateral wall [3, 4]. To achieve and maintain the unique composition of the endolymph, multiple energy-neutral ion transporters, such as Na^+^, K^+^, and 2Cl^−^ cotransporters (NKCCs), are active in the lateral wall cells, along with active transport of K^+^ by ion pumps, such as Na^+^, K^+^ ATPases, and voltage-gated K^+^ channels [3]. Dysfunction of the genes contributing to homeostasis of the cochlear endolymph, such as *ATP1A3* [5], *KCNQ1* [6], and *KCNE1* [7], leads to CAPOS (cerebellar ataxia, areflexia, pes cavus, optic atrophy, and sensorineural hearing loss) syndrome (MIM: 601338), Jervell and Lange-Nielsen syndrome (JLNS) 1 (MIM: 220400), and JLNS2 (MIM: 612347), respectively, which all have hearing loss as a major clinical feature. Inner-ear dysfunction, based on the failure to maintain endolymph homeostasis, has been proposed as “endolymphatic deafness” [8]. Although less clearly elucidated, Cl^−^ has also been recognized as important for proper cochlear function, since dysfunction of *CLIC5*, which mediates Cl^−^ efflux [9], or *SLC26A4*, a Cl^−^/I^−^ and Cl^−^/HCO_3_^−^ exchanger that mediates Cl^−^ influx [10], leads to the autosomal-recessive hearing loss conditions DFNB103 (MIM: 616042) and DFNB4 (600791), respectively.

Genetic analyses of known deafness genes [11–13] can be applied for diagnostic tests, while whole-exome sequencing (WES) is effective for discovering novel disease-associated genes [14, 15]. Using WES, we identified three variants of *SLC12A2*, which encodes Na^+^, K^+^, 2Cl^−^ cotransporter 1 (NKCC1), in three independent families with segregating hearing loss. We also identified *SLC12A2* variant in the fourth family. The importance of *SLC12A2* in hearing has previously been reported in *Mus musculus* [16–19], but not in humans. We conducted splicing assays and functional analysis of the identified variants *in vitro*, and the results support the pathogenicity of *SLC12A2* variants in humans.

## Results

### Clinical presentation of patients

**Fig 1:**
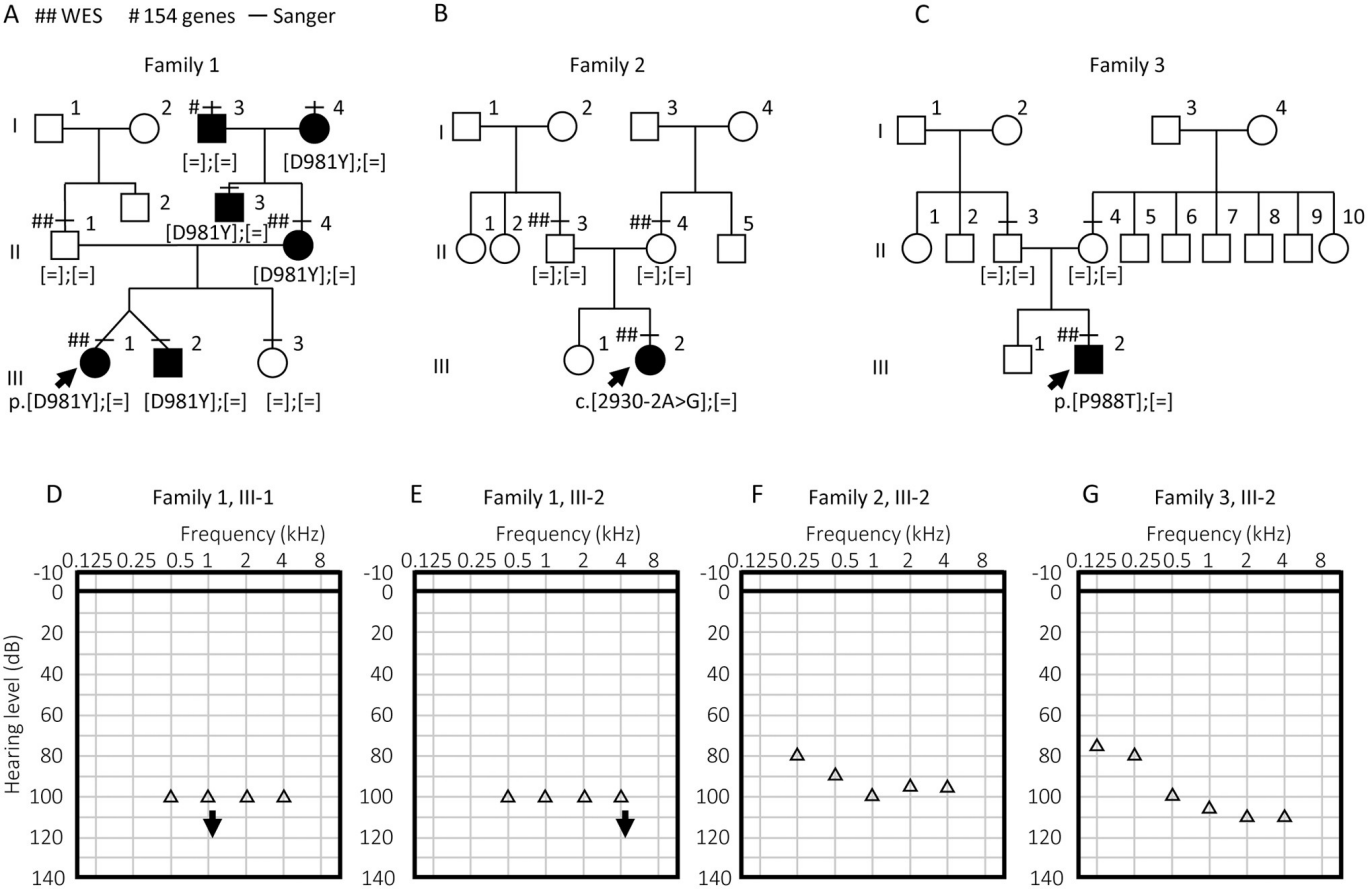
Subjects in this study. (A–C) Pedigrees of families 1 (A), 2 (B), and 3 (C). Arrows indicate probands. Double sharps (##), single sharps (#), and horizontal bars (–) indicate that individuals were subjected to WES, sequencing of deafness genes (154 genes), and/or Sanger sequencing for the specific variant (Sanger), respectively. *SLC12A2* (NM_001046.2) genotypes are indicated below the symbol representing each subject. [=] indicates that the position of an allele was identical to the reference sequence. (D–G) Audiograms measured by conditioned orientation reflex audiometry of the proband (D) and her twin brother (E) in family 1, the proband of family 2 (F), and the proband of family 3 (G). Open triangles indicate thresholds measured in both ears. Downward arrows indicate that the hearing level was undetectable at the respective frequency of sound.

In family 1, the proband was a 7-month-old female infant (Fig 1A, III-1). The proband and her twin brother (III-2) both presented with congenital, profound hearing loss (Fig 1D and 1E, S1A and S1B Fig). The family exhibited an autosomal-dominant inheritance mode. Hearing was normal in III-3 (S1C Fig), whereas II-3, II-4, I-3, and I-4 also showed congenital profound hearing loss (S1D–S1G Fig).

In family 2, the proband was a 1 year, 5-month-old female toddler (Fig 1B, III-2), who presented as a sporadic case of congenital, severe hearing loss (Fig 1F). The proband could not hold her head up or maintain a sitting position until age 10 months, indicating a minor motor developmental delay.

In family 3, the proband was a 1 year, 10-month-old male toddler (Fig 1C, III-2), who had congenital, profound hearing loss as a sporadic case (Fig 1G, S1H Fig). The proband could not hold his head up until 6 months old and only started walking alone at 1 year, 8 months, indicating borderline motor developmental delay.

CT of the temporal bones did not detect any anomaly of the inner, middle, or outer ears in any of the patients examined (family 1, III-1 and III-2; family 2, III-2; family 3, III-2). Visual inspection of the entire body, palpation of the head and neck, interview of affected individuals or their parents did not detect additional symptoms of multiple organs [20], global developmental delay [21], macrocephaly and epilepsy [22], or schizophrenia [23], all of which have been reported to be associated with *SLC12A2* variants. Body weight and height, motor and neuronal development, as well as behavior and cognition were within normal range.

Clinical presentation of family 4 is briefly described in the section of genomic analysis.

### Genomic analysis

Details of the three candidate pathogenic variants detected by genomic analysis are presented in Table 1, and a summary of the WES analyses is presented in S1 Table.

**Table 1:** *SLC12A2* variants detected in this study.

Family	Genome position (GRCh37: chr5)	Nucleotide change (NM_001046.2)	Amino acid change	Population databases*	REVEL	CADD	Effect on exon 21 acceptor site**	PhyloP100way	Conservation***
1	127512808	c.2941G>T	p.D981Y	None	0.536	31	−6.14%	8.727	12/12, 48/48, 29/30
2	127512795	c.2930–2A>G	Splice-site mutation	None	–	33	−41.22%	8.259	12/12, 48/48, 30/30
3	127512829	c.2962C>A	p.P988T	None	0.518	31	None	6.914	12/12, 48/48, 30/30

*Results from public population databases (dbSNP, 1000Genomes, ESP6500, ExAC, gnomAD, HGVD) and in-house databases (1,000 healthy Japanese individuals for family 1 and 2,600 Japanese family members with congenital brain anomaly or hearing loss for family 3).

**Results from Human Splicing Finder ver3.0.

***Identity of amino acid residues p.981D, 988P, and nucleotide c.2930–2A, in 12 primate, 48 mammal, and 30 vertebrate species.

REVEL, Rare Exome Variant Ensemble Learner; CADD, Combined Annotation Dependent Depletion (v1.4); PhyloP100way, 100 vertebrates Basewise Conservation by PhyloP.

**Fig 2:**
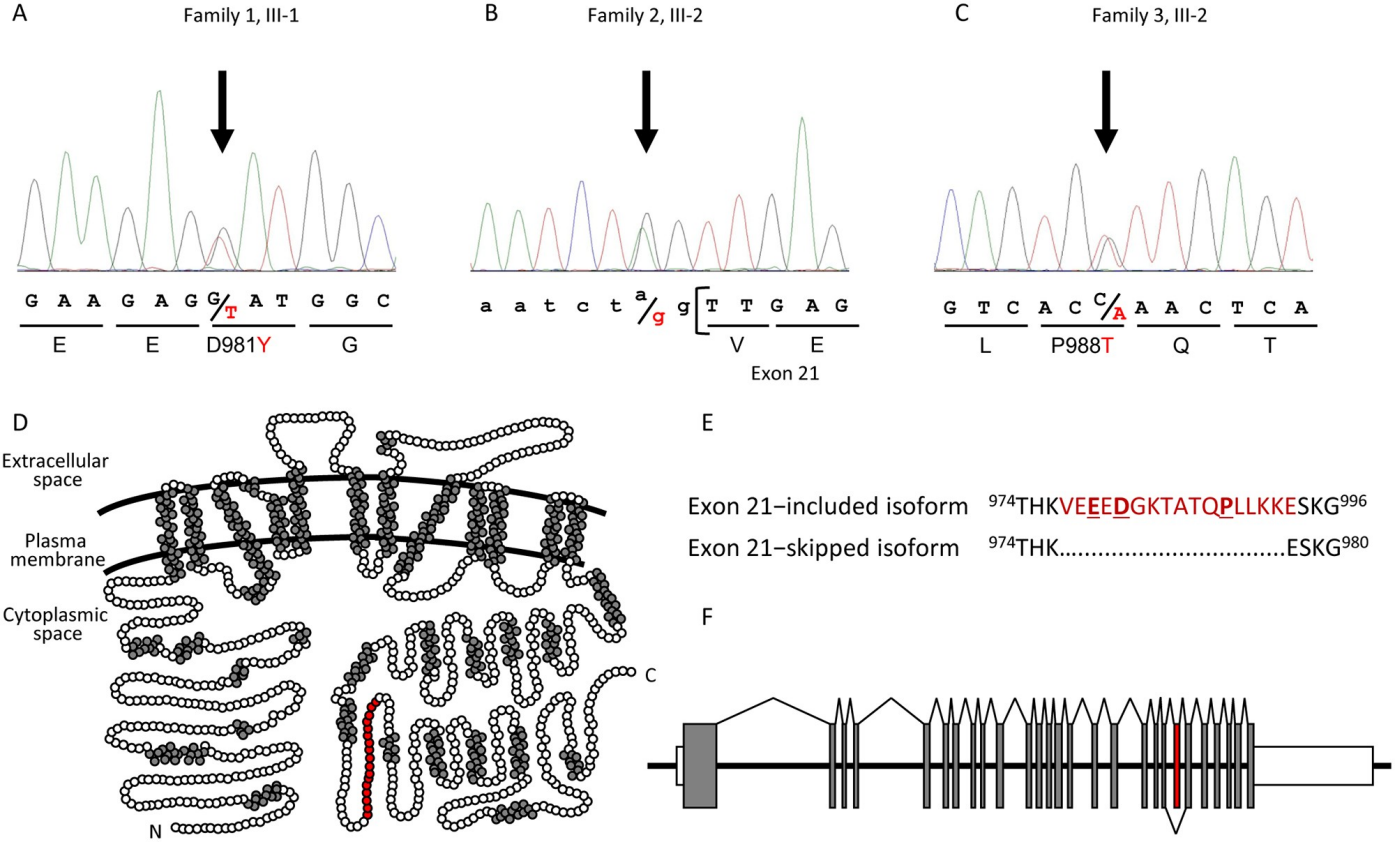
*SLC12A2* and the variants detected in this study. (A–C) Partial electropherograms showing heterozygous variants detected in the probands from families 1 (A), 2 (B), and 3 (C; reverse complementary sequence). (D) Schematic structure of SLC12A2 (GenBank: NP_001037.1, based on [34]). The plasma membrane is indicated by curved lines. Amino acid residues contributing to putative helical structures, and those encoded by exon 21, are shown by gray and red circles, respectively. (E) Partial amino acid sequences of the exon 21-included isoform (*top*) and exon 21-skipped isoform (*bottom*). Numbers indicate the positions of both ends of the protein region. The exon 21-encoded region is indicated in red. p.979E, 981D, and 988P are indicated in bold letters and underline. (F) Genomic structure of *SLC12A2*. Exons with protein-coding regions are shown by gray boxes. Exon 21 is indicated by a red box. Untranslated regions are shown by white boxes. Splice junctions are connected by lines, to indicate that both the exon 21-included (*top*) and -skipped (*bottom*) variants are transcribed.

For family 1, based on trio WES analysis, including samples from the proband (III-1) and the parents (II-1, II-4), we selected 38 coding and splice-site variants with low minor allele frequencies in population databases that co-segregated with hearing loss (see Materials and methods for details, S4 Table). To narrow down these candidate variants, genes were classified into three categories and prioritized for analysis, as described in the Materials and Methods. No Tier 1 genes (previously identified deafness genes in humans; S2 Table) with autosomal dominant inheritance were among the candidate variants, and *SLC12A2* [16–19] was the only Tier 2 gene (genes associated with hearing loss in mouse models; S3 Table) among the variants. Sanger sequencing identified a heterozygous variant of *SLC12A2* (c.2941G>T), resulting in a D981Y amino acid change in I-4, II-3, II-4, III-1, and III-2, who had hearing loss, but not in the other subjects tested (Figs 1A and 2A, S2A Fig), indicating co-segregation of the variant with hearing loss, with the exception of I-3, who had hearing loss, but did not carry this variant. To further investigate the possible cause of hearing loss in I-3, we conducted sequencing analysis targeting coding exons of 154 deafness genes and candidate deafness genes [11] (S7 Table) in this individual. The results indicated that he carried a heterozygous variant of *MYH19* (NM_002473:c.5765+9C>G, rs201008102) associated with DFNA17 with uncertain pathogenicity; however, neither II-4 nor the proband (III-1) carried this variant, indicating that it did not co-segregate with hearing loss in the family. Hence, the cause of hearing loss in I-3 is unknown at present. Overall, although we cannot exclude other possibilities, such as linkage disequilibrium of the *SLC12A2* variant with a possible cryptic pathogenic change, or variants in unknown deafness genes, also present in I-3, which could be responsible for the disease, the *SLC12A2* variant was considered the best candidate for association with hearing loss.

For family 2, trio WES analysis identified five candidate variants in four genes (one variant each of *SLC12A2*, *CASC1*, and *C5orf51*, and 2 variants of *SZT2*, S5 Table). No Tier 1 gene was among the identified variants, and *SLC12A2* was the only Tier 2 gene. Sanger sequencing confirmed that the heterozygous c.2930‒2A>G variant of *SLC12A2* in the proband reflected a *de novo* mutation (Fig 2B, S2B Fig). Human Splicing Finder ver3.0 [24] generated a prediction value for the altered acceptor site of −41.22%, well beyond the threshold (−10%), suggesting that inclusion of exon 21 is disrupted by the variant (Table 1). In this family, parenthood of the proband was investigated by counting the numbers of variants she inherited. We found that 98 and 103 of 217 protein-affecting variants with MAF < 0.003 appeared to be derived from the father (II-3) and mother (II-4), respectively, confirming their parenthood (S3A Fig).

For family 3, singleton WES analysis of the proband focusing on Tier 1 and 2 genes detected candidate heterozygous variants of *TECTA* (NM_005422.2: c.4495G>C, MIM: 601543) [25, 26] among the Tier 1 genes and *SLC12A2* and *ACAN* (NM_001135.3: c.83C>G) among the Tier 2 genes. *ACAN* has been associated with hearing loss in *M*. *musculus* [27]; however, it is associated with other diseases in humans (short stature and advanced bone age, with or without early-onset osteoarthritis and/or osteochondritis dissecans (MIM: 165800) and spondyloepiphyseal dysplasia (MIM: 608361 and 612813)). Sanger sequencing revealed that the *TECTA* and *ACAN* variants detected in the proband (III-2) appeared to be derived from the father (II-3) and the mother (II-4), respectively (S4A and S4B Fig, S6 Table). The both parents had normal hearing; thus these variants are unlikely to be associated with hearing loss. The heterozygous c.2962C>A (p.P988T) variant of *SLC12A2* was confirmed to be a *de novo* variant by Sanger sequencing, and considered the best candidate for association with hearing loss (Fig 2C, S2C Fig). In this family, parenthood was studied by comparison of two short tandem repeat (STR) markers among the proband and his parents, since the proband was subjected to singlet WES analysis and it was not possible to count the numbers of variants inherited from the parents. STR markers detected in the proband appeared to be derived from the parents (II-3 and II-4), confirming their parenthood (S3B Fig).

These three *SLC12A2* variants were considered the best candidates for causing hearing loss in the families. The wild-type (WT) sequences at the positions of each variant are highly conserved among 90 vertebrate species, and none of the variants were present in public population databases (Table 1).

In parallel to WES analysis, we conducted sequencing analysis focused on 154 deafness genes and candidate deafness genes (S7 Table) to subjects with hearing loss. In the analysis, a sporadic case was found to have *de novo* c.2935G>A (p.E979K) variant of *SLC12A2* (S5A, S5E–S5G Fig). The proband was a 7 months-old female infant (S5A Fig, III-2), who had congenital, severe hearing loss (S5B and S5C Fig). The position of E979 appeared to encode acidic glutamate or aspartate among vertebrate species; thus, polarity of the amino acid residue at this position was conserved. The proband did not show a motor developmental delay or inner ear malformation. STR markers detected in the proband appeared to be derived from the parents (II-1 and II-4), confirming their parenthood (S5H Fig).

Genomic analysis of human population databases revealed that *SLC12A2* is intolerant of functional variation (the z score for the constraint metric for missense variants is positive (2.4), and the probability of *SLC12A2* being loss of function-intolerant (pLI) is ≥ 0.9 (0.96), according to gnomAD version 2.1 [28]). These scores support the possibility that the missense variants detected in families 1 and 3 alter the function of SLC12A2.

### Conservation of exon 21-encoded amino acid residues

Intriguingly, all four *SLC12A2* variants mapped to exon 21 or its 3’-splice site (Fig 2D and 2E). Among the 27 exons within the *SLC12A2* genomic DNA (Fig 2F), exon 21 consists of 48 base pairs and encodes 17 amino acid residues (including partial codons for two residues at each end) in the long cytoplasmic stretch of the SLC12A2 protein, after the 12 transmembrane regions. There are two known alternatively spliced transcripts of human *SLC12A2*: an exon 21-included transcript encoding a long isoform (NP_001037.1), and an exon 21-skipped transcript encoding an in-frame, 16 residue truncated isoform (NP_001243390.1) (Fig 2E and 2F). Therefore, the three missense variants were expected to affect the function of the long isoform, and the splicing variant was anticipated to lead to the excision of exon 21, leading to increased production of the truncated isoform.

Although residues E979, D981, and P988 are well conserved among SLC12A2 orthologs in vertebrate species (Table 1, S5D Fig), multiple alignment of the eight SLC12A family proteins (S6 Fig) revealed that the residues encoded by exon 21 are unique to SLC12A2 and are not conserved, even in the most closely related proteins in this family, SLC12A1 (also known as NKCC2) and SLC12A3 (NCC) [29]. This finding suggests that, during the molecular evolution of SLC12A family members, exon 21 encoded a peptide sequence that conferred a specific functional characteristic to the protein.

### Analysis of *SLC12A2* exon 21 splicing *in vitro* and in mammalian tissues

**Fig 3:**
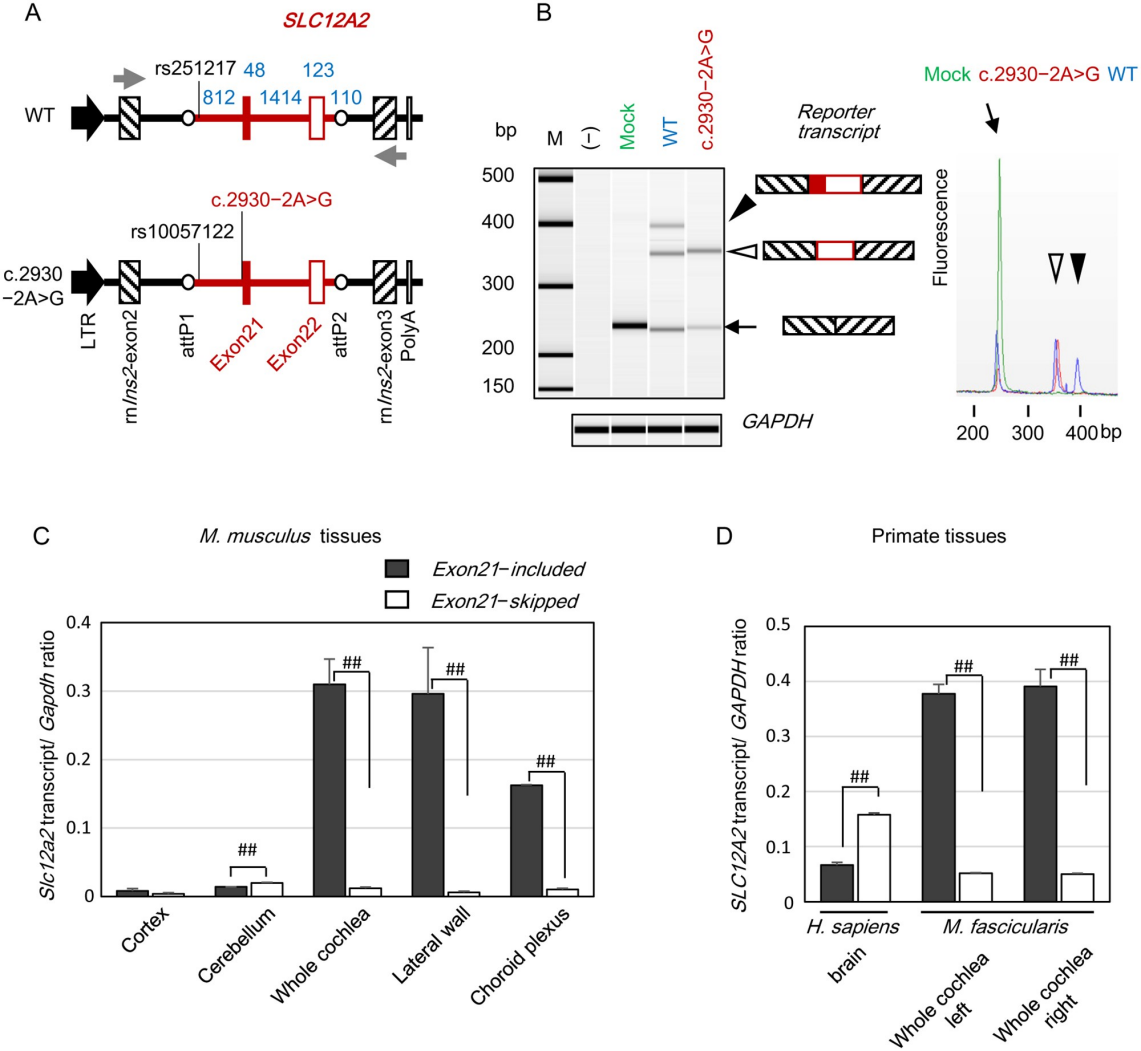
Splicing analysis of *SLC12A2* exon 21 and expression levels of *SLC12A2* transcripts in mammalian tissues. (A) Minigene structures. Rous sarcoma virus long terminal repeat (LTR, black arrows), rat insulin-2 (*rnIns2*, boxes with slashes), BP clonase site attP1/P2 (open circles), and partial genomic region of WT *SLC12A2* or *SLC12A2* with the splice-site mutation (c.2930–2A>G) (red line) including exons 21 (filled red box) and 22 (open red box) are shown. Numbers in blue indicate the nucleotide length (bp) of each *SLC12A2* genomic region. Small gray arrows indicate positions of primer sets for RT-PCR (S8 Table). Positions of SNPs and the splicing variant of *SLC12A2* are also indicated. (B) *Left*, RT-PCR (N = 3) to detect reporter transcripts in HEK293T cells transfected with the respective vectors. The image of the RT-PCR products was generated using a BioAnalyzer 2100. The size of each reporter transcript is indicated by an open or filled arrowhead, or small arrow. M, 100 bp ladder marker; (–), no transfection; mock, empty vector. *Right*, Partial electropherograms of reporter transcripts. Schematics of each transcript are indicated by arrowheads or small arrows. (C,D) Quantitative RT-PCR (qRT-PCR) to measure the expression levels of the *Slc12a2* exon 21-included and -skipped transcripts in *M*. *musculus* tissues (N = 3) (C) and in human brain and whole cochlea from *M*. *fascicularis* (D). ##, *p* < 0.005.

To investigate whether the c.2930‒2A>G (family 2) variant affects inclusion of exon 21 of *SLC12A2*, reporter minigenes were constructed by inserting the genomic region, including exons 21 and 22 of the gene, with or without the splicing variant, into the pSpliceExpress vector [30] (Fig 3A). Sanger sequencing revealed that the WT and candidate splicing variant alleles harbored additional variants, c.2930‒733A>G (rs251217) and c.2930‒734T>C (rs10057122), respectively (Fig 3A). As rs10057122 and rs251217 are common variants (35.2% and 36.4% in 1000Genomes, respectively), they were considered non-pathogenic and unlikely to substantially affect splicing of exon 21.

When HEK293T cells were transfected with the vector harboring the WT allele, RT-PCR detected three PCR products as reporter transcripts (Fig 3B). The longest product corresponded to the reporter transcript including both exon 21 and exon 22 of *SLC12A2* (filled arrowhead in Fig 3B), a shorter PCR product corresponded to the reporter transcript without exon 21 (open arrowhead in Fig 3B), and the shortest PCR product corresponded to a transcript containing neither exon 21 nor exon 22 (arrow in Fig 3B). The shortest product was also detected in the cells transfected with empty vector (mock), indicating that it was generated from the vector construct. When cells were transfected with vector harboring the c.2930‒2A>G variant allele, the longest PCR product disappeared, whereas the two shorter PCR products remained detectable. These results strongly suggest that the c.2930‒2A>G variant disrupts the inclusion of exon 21 in the *SLC12A2* transcript, resulting in expression of only the exon 21-skipped transcript from the variant allele.

We next explored whether both the exon 21-included and -skipped alternative transcripts of *SLC12A2* were expressed in cochlear tissues. Quantitative RT-PCR (qRT-PCR) demonstrated that expression ratios of exon 21-included/skipped transcripts were dramatically different among mammalian tissues. In whole cochlea and lateral wall, exon 21-included transcript was expressed at significantly higher levels than the exon 21-skipped transcript (Fig 3C, S7A Fig), with ratios of exon 21-included/skipped transcripts similar to that in the choroid plexus; in these tissues, levels of the exon 21-included transcript were significantly higher than those in the cortex and cerebellum (Fig 3C, S7A Fig). Further, expression of the exon 21-included transcript was significantly lower than that of the exon 21-skipped transcript in the cerebellum, with no significant difference detected in the cortex (Fig 3C, S7A Fig). Similar to the results using *M*. *musculus* tissues, exon 21-included transcript levels in cochleae dissected from a 3-year-old male *Macaca fascicularis* were significantly higher than those of the exon 21-skipped transcript, while the exon 21-included transcript was expressed at significantly lower levels in *Homo sapiens* brain (Fig 3D, S7B Fig).

Consistent with the qRT-PCR results, RT-PCR analysis detected only exon 21-included transcript in whole cochlea and lateral wall samples from *M*. *musculus*, and predominant expression of the exon 21-included transcript in cochlea from *M*. *fascicularis* (S8A and S7B Figs). Predominant to exclusive exon 21-included transcript expression was also observed in the choroid plexus, eye, spleen, liver, intestine, kidney, and ovary of *M*. *musculus*. By contrast, both transcripts were detectable in the cortex and cerebellum of *M*. *musculus*, as previously reported [31]. Similarly, both transcripts were detectable in *H*. *sapiens* brain, as previously reported [32]. Overall, expression patterns of *Slc12a2* in mammalian cochlea and central nervous systems determined by RT-PCR resembled those determined by qRT-PCR (summarized in S9 Fig). Junction expression of *SLC12A2* in various *H*. *sapiens* tissues, determined as part of the Genotype-Tissue Expression Project (GTEx) [33], also demonstrated that the exon 21-skipped transcript (ETS00000343225.4) is detectable at high levels in the central nervous system (S10 Fig). These data indicate that mammalian cochleae almost exclusively express the exon 21-included transcript of *SLC12A2*, and that exon 21 skipping is a minor event in the mammalian cochlea and tissues other than those of the central nervous system.

### Functional consequences of recombinant (rec) SLC12A2 variants

**Fig 4:**
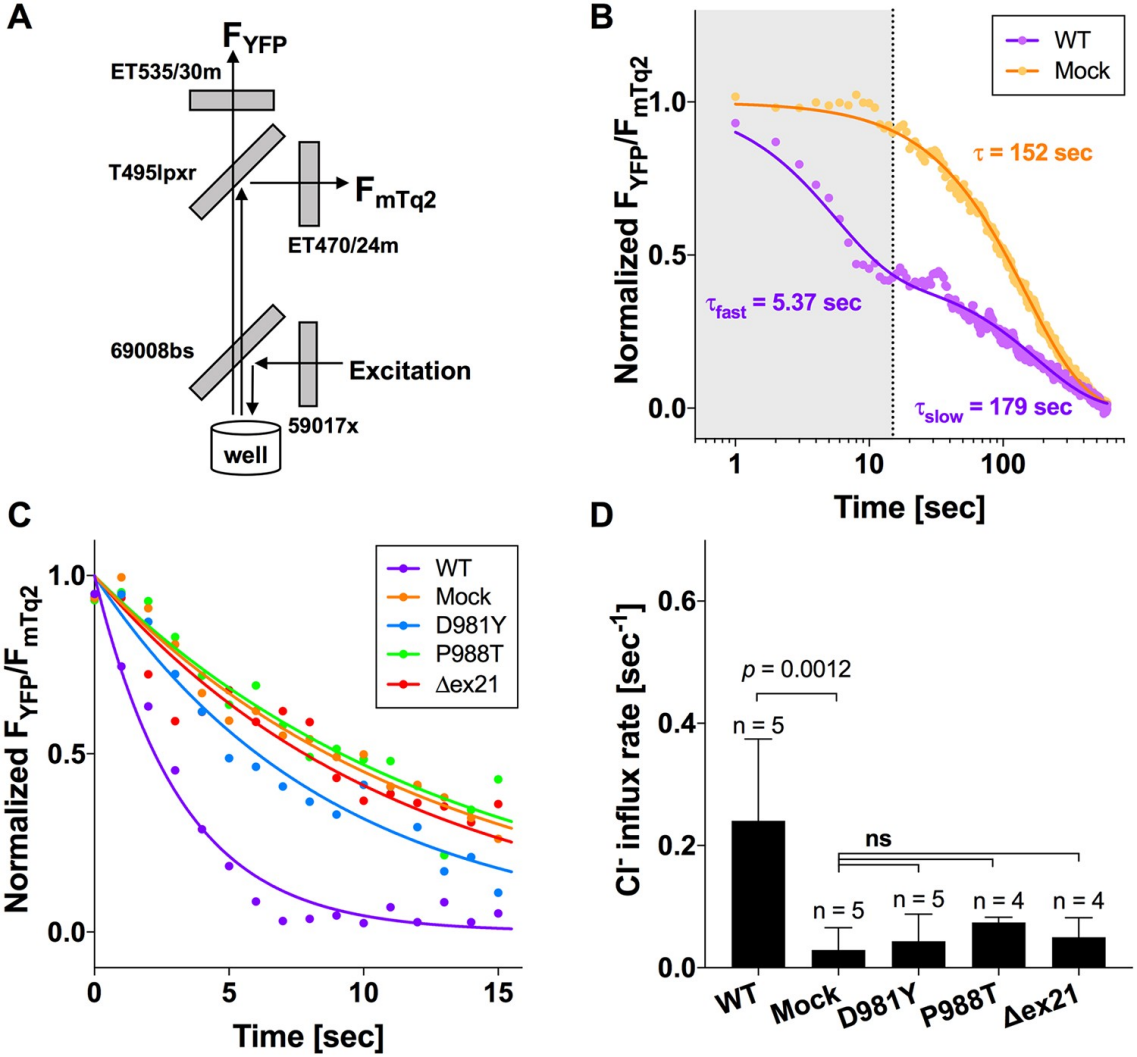
Cl^−^ influx assay of the recSLC12A2 constructs *in vitro*. (A) Schematic representation of the optical configuration used for simultaneous measurements of YFP and mTurquoise2 (mTq2) fluorescence (F_YFP_ and F_mTq2_), showing band-pass filters and dichroic mirrors. (B) Cl^−^ influx measured in WT recSLC12A2- (purple) and empty vector-derived YFP (mock, orange)-expressing cells. Solid lines indicate single (for mock) and double (for WT recSLC12A2) exponential curve fitting. A 15 sec time window (area shaded in gray) was used to calculate recSLC12A2-mediated Cl^−^ influx. (C) Representative recordings from cells expressing each recSLC12A2 variant are shown in the respective colors. Solid lines indicate single exponential curve fits from which Cl^−^ influx rates were calculated. (D) Summary of the Cl^−^ influx assay. One-way ANOVA and Tukey-Kramer multiple comparison tests were performed to calculate the adjusted *p* values. Error bars represent SD. ns, not significant (*p* ≥ 0.05); Δex21, exon 21-encoded region truncated isoform.

To determine the effects of the *SLC12A2* variants on Na^+^, K^+^, 2Cl^−^ cotransport activity, we established HEK293T cell lines that heterologously expressed WT, mutated (D981Y or P988T), or exon 21 truncated (corresponding to p.977_992del) recSLC12A2 proteins in a doxycycline-dependent manner. Cl^−^-sensitive yellow fluorescent protein (YFP) was attached to the amino-terminus of each of these SLC12A2 constructs so that the ion transport activity of WT, mutated, and truncated recSLC12A2 proteins could be assessed by monitoring Cl^−^ influx [34]. mTurquoise2 (mTq2) was constitutively expressed in these cell lines as a reference fluorescent protein. As cell suspensions were used for our Cl^−^ influx assay, simultaneous measurements of YFP fluorescence (F_YFP_) and reference mTq2 fluorescence (F_mTq2_) allowed correction for fluctuations in F_YFP_ between measurements that could be attributed to changes in the number of cells in the light path (Fig 4A). Prior to measurement, all cell lines were incubated in a low Cl^−^-containing solution, which is essential for activating SLC12A2 [35, 36]. The Cl^−^ influx assay was initiated by the injection of a high-Cl^−^ buffer. WT recSLC12A2-expressing cells showed biphasic Cl^−^ influx kinetics, which was in stark contrast to the monophasic kinetics of empty vector-derived YFP-expressing cells (mock, Fig 4B). The monophasic Cl^−^ influx kinetics that were apparent for the YFP-expressing cells indicated that the HEK293T cells retained endogenous Cl^−^ influx activity. The time constant for this endogenous Cl^−^ influx resembled the slower time constant (τ_slow_) observed for WT recSLC12A2-expressing cells (152 sec vs. 179 sec, Fig 4B). A previous study also reported biphasic Cl^−^ influx kinetics in SLC12A2-expressing cells, likely ascribable to deactivation of SLC12A2 upon Cl^−^ influx [36]. Thus, we assessed the ion transport activity of recSLC12A2 and its variants, based on the Cl^−^ influx rates determined within 15 sec after the injection of the high-Cl^−^ solution (Fig 4C). We found that the Cl^−^ influx rate for each of the variants (D981Y and P988T) and the truncated recSLC12A2-expressing cells was significantly lower than that of WT recSLC12A2-expressing cells, with values statistically indistinguishable from those of YFP-expressing cells (Fig 4D), suggesting that the Na^+^, K^+^, 2Cl^−^ cotransport activity of SLC12A2 was significantly compromised in cells expressing each of these variants.

**Fig 5:**
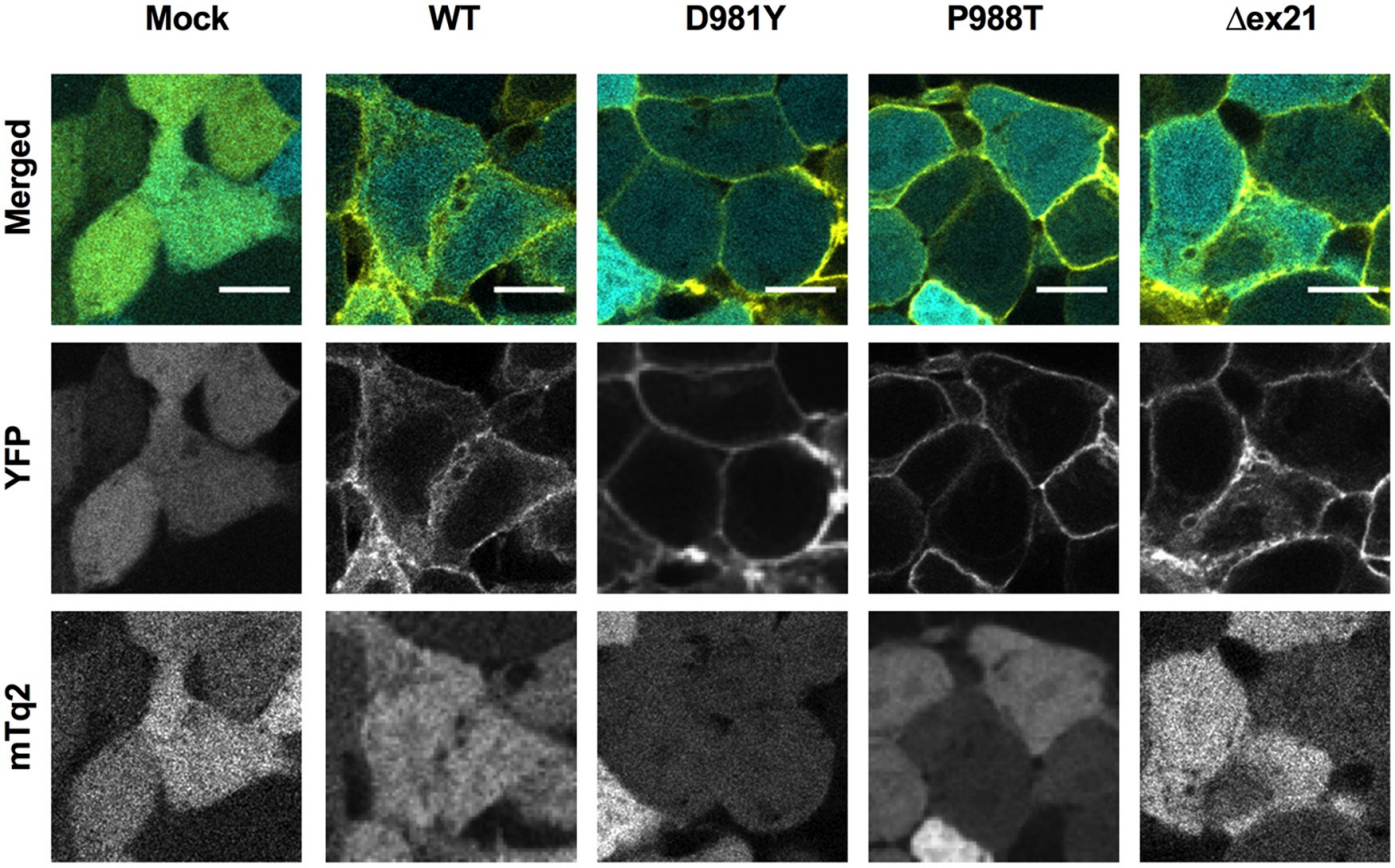
Plasma membrane localization of the recSLC12A2 constructs in HEK293T cells. *Top*, Cellular localization of the recSLC12A2 constructs was assessed by observing the fluorescence of YFP (yellow) attached to the amino-terminus of each SLC12A2 construct. The signals were merged with those of cytosolic mTq2 (cyan) co-expressed with each construct to identify the cell boundaries. *Middle* and *bottom*, images of YFP (*middle*) and mTq2 (*bottom*) channels are shown in gray. Scale bars, 10 μm.

The exon 21-encoded region of SLC12A2 contains a dileucine sorting motif, which is important for intracellular/membrane trafficking [37]. To gain further insights into the dysfunction of these recSLC12A2 variants, we examined the subcellular localization of the recSLC12A2 proteins in the HEK293T cells used for the Cl^−^ influx assay. As shown in Fig 5, the WT, variant, and truncated recSLC12A2 proteins all appeared to be successfully targeted to the plasma membrane. Therefore, the compromised transport activities of the recSLC12A2 constructs could be attributed to alterations in their function and not to impaired membrane targeting.

### SLC12A2 signals in *M*. *fascicularis* cochlea

**Fig 6:**
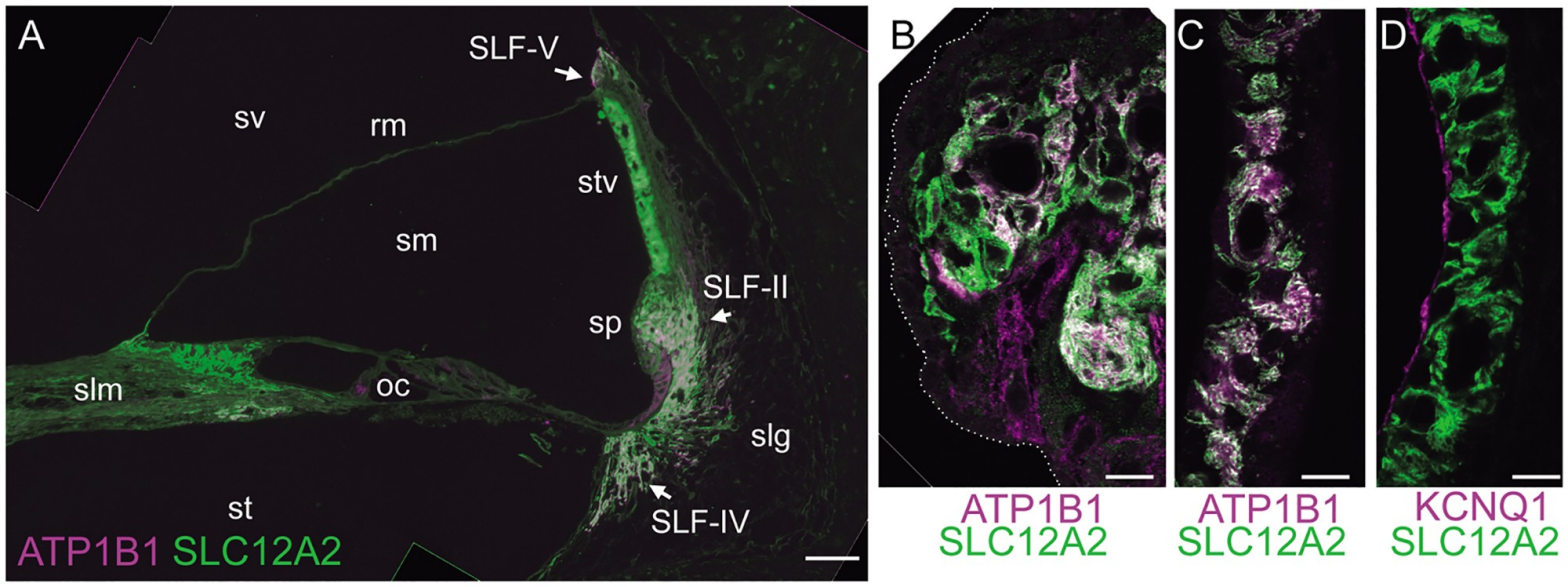
Distribution of SLC12A2 signals in the *M*. *fascicularis* cochlea. Paraffin sections of cochlear tissue from adult *M*. *fascicularis* (N = 3) were incubated with rabbit antiserum against SLC12A2 (green) and monoclonal antibody against ATPlBl (magenta) (A–C) or KCNQl (magenta) (D). (A) Overall image, showing the distribution of SLC12A2 in the cochlea. Scale bar, 50 μm. (B–D) Magnified image of the spiral prominence (B) or stria vascularis (C,D) immunostained with the indicated antibodies. Dotted line in (B) indicates the apical surface of the epithelial cells of the spiral prominence. Scale bars, 10 μm. oc, organ of Corti; rm, Reissner’s membrane; SLF-II, -IV, and -V, spiral ligament fibrocyte type II, IV, and V, respectively; slg, spiral ligament; slm, spiral limbus; sm, scala media; sp, spiral prominence; st, scala tympani; stv, stria vascularis; sv, scala vestibuli.

Immunohistochemical study using anti-SLC12A2 antibody performed on *M*. *fascicularis* cochleae demonstrated positive SLC12A2 signals in the stria vascularis, the spiral prominence, and parts of the spiral ligament (Fig 6A, S11 Fig). SLC12A2-positive cells in the spiral ligament were also positive for the type II/IV/V fibrocyte marker, ATP1B1 (also referred to as Na^+^, K^+^-ATPase β1; Fig 6A). These results demonstrate that the distribution of SLC12A2 in the *M*. *fascicularis* cochlea is essentially the same as reported for guinea pig, gerbil, and rat [38–40]. The spiral limbus and a small area of Reissner’s membrane connected to the spiral limbus were also positive for SLC12A2 (Fig 6A). No SLC12A2 signal was detected in the organ of Corti or epithelial cells of the spiral prominence (Fig 6A and 6B). In the strial marginal cells, SLC12A2 signals colocalized with those of ATP1B1 (Fig 6C) and were visible at the basolateral membrane, but not the apical surface, as shown by the absence of colocalization with KCNQ1, a specific marker for the apical membrane of strial marginal cells (Fig 6D. S11B Fig).

## Discussion

### Association of *SLC12A2* with hearing loss in humans

In this study, we identified four candidate variants of *SLC12A2* in four families with sensorineural hearing loss. *In vitro* studies demonstrated that a splice-site variant at the exon 21 acceptor site (c.2930‒2A>G) inhibited inclusion of exon 21 and increased generation of an exon 21-skipped transcript from the variant allele. Further, the exon 21-skipped transcript was expressed at very low levels in mammalian cochleae. HEK293T cells expressing recSLC12A2 proteins carrying each of the three variants showed significantly decreased Cl^−^ influx activity. Moreover, we observed abundant SLC12A2 signals in the basolateral membrane of strial marginal cells and lateral wall fibrocytes in non-human primate cochlea, a distribution identical to that reported for rodent cochleae [16], suggesting a conserved role for SLC12A2 in homeostasis of the endolymph by recycling K^+^ from the perilymph to the stria vascularis in mammalian cochlea, including in humans [3, 4]. The congenital, severe-to-profound hearing loss in our patients is consistent with the phenotype of *Slc12a2*-deficient *M*. *musculus* [16–19]. In addition to hearing loss, two of our patients (III-2 in family 2 and III-2 in family 3) presented with motor developmental delay. Since the patients with motor developmental delay caught up with expected milestones later, the feature was considered to be comorbid with vestibular impairment that could be compensated, unlike muscle, bone, or brain disorders, which cannot be compensated. Patients with hearing loss and symptoms derived from inner ear dysfunction have been categorized as having nonsyndromic hearing loss; for example, DFNA9 (MIM: 601369), DFNA11 (601317), and DFNB4 (600791) all exhibit vestibular abnormalities as a major clinical feature associated with hearing loss and are categorized as nonsyndromic hearing loss. Therefore, we propose that mutations in *SLC12A2* are associated with autosomal-dominant nonsyndromic hearing loss.

SLC12A2 is the entry site for Na^+^, K^+^, and 2Cl^−^ from the intrastrial space to the strial marginal cells in the cochlear lateral wall [4]. *Slc12a2-*deficient *M*. *musculus* show loss of scala media [16–19], while homozygous *Slc12a2* deficiency results in loss of endolymph volume, collapse of the otic vesicles with functional sensory hair cells, and a vestibular disorder phenotype in *Danio rerio* larvae [41]. The disrupted Cl^−^ influx in cells expressing the three variants reported here strongly suggests that the mechanisms underlying hearing loss include, at least in part, impaired production of endolymph secreted from the strial marginal cells, attributable to decreased activity of the Na^+^, K^+^, 2Cl^−^ cotransporter.

Intriguingly, several reports have argued that *SLC12A2* is associated with other clinical features; for example, a heterozygous p.V1026Ffs*2 variant or a splice variant, affecting both exon 21-included and -skipped transcripts, might be associated with malformation of multiple organs without hearing loss [20], or global developmental delay [21], respectively. Further, missense variants mapping to exon 1 [22] or 17 [23] may increase the risk of morphological or functional disorders of the central nervous system. In addition, *Slc12a2*-deficient *M*. *musculus* exhibit hearing loss, vestibular defects, and additional symptoms, including small body size, hyper-excitability, and male infertility (reviewed in [29]). These additional phenotypes were not observed in the probands or in the other family members with hearing loss in this study. These differences could be attributable to the fact that all the candidate variants identified in this study are predicted to affect exon 21, without degeneration of the transcript. We speculate that the variants affecting the exon 21-encoded region without protein degeneration not only deteriorate cotransporter activity, but also affect an unrecognized function of SLC12A2, both of which are critical for hearing. This possibility awaits further investigation before firm conclusions can be drawn.

### Difference in the inheritance pattern of hearing loss associated with *SLC12A2* variants between humans and *M*. *musculus*

A discrepancy was noted between the results obtained with animal models and observations in our patients in the inheritance pattern of the hearing loss phenotype; our data indicate that heterozygous variants of *SLC12A2* are associated with hearing loss in humans, whereas *Slc12a2* is responsible for autosomal-recessive hearing loss in *M*. *musculus* [16–19]. One possible explanation for this difference is that the functional SLC12A2 derived from a single allele is not sufficient for normal hearing in humans, whereas it is sufficient in *M*. *musculus*; however, based on the assumption that *SLC12A2* is responsible for monogenic disease, the idea that haploinsufficiency of *SLC12A2* is associated with hearing loss is inconsistent with the fact that multiple loss-of-function variants of *SLC12A2* have been detected in subjects with multiorgan dysfunction and normal hearing (p.V1026Ffs*2) [20] or registered in ExAC and gnomAD databases (such as p.G55Vfs*5, Y257Cfs*10, Q435*, R482*, P988Rfs*4, K1007Sfs*29, Q1099Ifs*3, Y1111*, and R1133*) [28, 42], although the allele frequencies of all the loss-of-function variants are very low (the highest MAF among loss-of-function variants in gnomAD is 6.49 × 10^−5^ for the p.G55Vfs*5 variant). All those nonsense and indel variants, including p.P988Rfs*4 in exon 21, are predicted to result in degeneration of the transcript by nonsense-mediated mRNA decay [43]. Therefore, although the reason for intolerance to loss-of-function variants of *SLC12A2* in population databases remains unclear, haploinsufficiency does not appear to adequately explain the molecular mechanism underlying hearing loss caused by *SLC12A2*, without taking additional modifier factors into consideration [44].

An alternative explanation for the difference between human patients and *Slc12a2*-deficient mouse models is that variants affecting the exon 21-encoding region without degeneration of the transcript may alter as yet unidentified roles of SLC12A2, in addition to disrupting ion transport activity, leading to hearing loss as gain-of-function or dominant negative effect. It is intriguing to note that the expression level of the exon 21-skipped *SLC12A2* transcript was significantly lower than that of the exon 21-included transcript in mammalian cochlear tissues, unlike previous reports in brain tissues [31, 32]. Our *in vitro* experiments suggest that the c.2930–2A>G variant leads to expression of the exon 21-skipped transcript and increased overall expression levels of this exon 21-skipped transcript in the cochlea. The exon 21-skipped or altered isoform of SLC12A2 may have roles in addition to ion transport that are beneficial for the central nervous system, but unfavorable for the cochlea. *Slc12a2*-knockout mice have been generated by elimination of both the exon 21-included and -skipped isoforms, by disruption of exon 9 [16], exon 6 [18], or exons 9–11 [19]. All these knockout strains are reported to result in degeneration of both *Slc12a2* transcripts. Another *Slc12a2*-deficient mouse, with the c.2958dupA variant in exon 21 [17], results in absence of Slc12a2 signals in the cochlea, where the exon 21-included transcript is predominantly expressed. By contrast, the four variants found in our patients are considered to result in expression of the transcript without degeneration of the variant allele, or increased expression of the exon 21-skipped transcript, in the case of the c.2930–2A>G variant. Therefore the consequences of these variants would be expected to differ fundamentally from those reported for previous mouse strains. Hence, to verify the role of the exon 21 variants of *SLC12A2* in hearing loss, generation and investigation of animal models carrying the equivalent variants is required.

The discrepancies between this study and a previous report demonstrating that the exon 21-skipped SLC12A2 isoform retains K^+^ influx activity comparable to the exon 21-included SLC12A2 isoform, determined using Rb^+^ in MDCK cells [32], may reflect methodological differences in the measurement of the activity of the exon 21-skipped SLC12A2 isoform.

### Role of the exon 21-encoded region of SLC12A2

One possible mechanism of hearing loss by variants affecting exon 21 could be that they exert a dominant negative effect, in addition to resulting in decreased Cl^−^ influx activity. SLC12A2 and other proteins in the SLC12A family are thought to reside on the plasma membrane as homodimers [45–47] that form via association of the two carboxy-terminal domains [36, 47]. Whether the WT and variant and SLC12A2 molecules could generate a heterodimer, and thereby alter ion transport activity relative to the WT homodimer, warrants investigation in the future.

Another possible mechanism that could underlie hearing loss caused by the variants affecting exon 21 is that they may affect targeting of SLC12A2 to the basolateral membrane [37]. If variants affecting exon 21 alter the asymmetric distribution of properly functioning SLC12A2 on the strial marginal cells, this would affect its ability to secrete K^+^ into the endolymph; however, the choroid plexus, which predominantly expresses the exon 21-included variant, exclusively harbors SLC12A2 in the apical microvilli [48], indicating that asymmetric distribution is not fully dependent on the exon 21-encoded region. Whether targeting of SLC12A2 to the apical or basolateral membrane in the strial marginal cells is affected by variants in the exon 21-encoded region of SLC12A2 will be investigated in future experiments.

A third possible explanation for the effects of the exon 21 variants is that they may influence more complex properties of the strial marginal cells, through interaction with other molecules. *In vitro* studies have shown that SLC12A2 can be positively or negatively regulated through interaction of its carboxy-terminal region with the K^+^ and Cl^−^ cotransporters SLC12A7 (KCC4) [49] and SLC12A9 (CIP1) [50], respectively. Intriguingly, deficiency of *Slc12a7* causes progressive hearing loss in *M*. *musculus* [51]. Identification of partners that interact with the exon 21-encoded region of SLC12A2 may provide insight into the tissue-specific properties of the ion transporter isoforms, as well as the molecular pathology of hearing loss associated with *SLC12A2* variants.

The limitations of this study are as follows: 1) the small number of families investigated; additional cases and families, as well as careful clinical follow-up, are required to prove conclusively that *SLC12A2* is associated with nonsyndromic hearing loss; 2) lack of an animal model for investigation of the specific variants identified in this study; a mouse model carrying the *Slc12a2* variant equivalent to those identified in our patients should be generated and their phenotypes should be studied; 3) molecular effect of the variants on exon 21 is only partially explained by *in vitro* analysis; additional studies including transporter activity of the hetero dimers between WT and each variant, plasma membrane distribution in polarized cells, and identification of molecules associated with the exon 21-encoded region should be carried out.

In conclusion, whole-exome analysis or sequencing analysis targeting 154 deafness genes of four families with hearing loss identified *SLC12A2* as a novel candidate deafness gene. The molecular mechanism of hearing loss could include dysfunction of the cotransporter activity of SLC12A2 caused by the exon 21 variants, and may reflect other affected roles of the exon 21-encoded region. Future studies of the molecular regulatory mechanism of splicing of exon 21, and molecules associated with the exon 21-encoded region, may lead to a more precise understanding of the function of SLC12A2 in the inner ear, as well as of mechanisms underlying hearing loss, and provide insight for future development of drugs to treat hearing loss related to SLC12A2 dysfunction.

## Materials and methods

### Ethics statement

This study was approved by the institutional ethics review board at the National Hospital Organization Tokyo Medical Center (approval number: R1-0703009), the ethics committee at the at the National Hospital Organization Mie Hospital (R1-0703009), the ethics committee at the National Center for Child Health and Development (661), the ethics committee at the RIKEN Center for Integrative Medical Sciences (YokohamaH17-16(27), YokohamaH24-15(3)), and the ethics committee at the Tokyo Medical and Dental University (O2015-503). Studies were conducted only after patients or their parents signed informed consent to participate. For *in vitro* experiments, all procedures were approved by the Institutional Safety Committee for recombinant DNA Experiments at the National Hospital Organization Tokyo Medical Center and the Institutional Biosafety Committee at Northwestern University. Collection of animal tissue samples was approved by the Institutional Animal Care and Use Committee at the National Hospital Organization Tokyo Medical Center.

### Subjects

Medical histories and clinical information were obtained from probands and family members, when possible, by experienced otologists with the expertise in pediatric hearing loss (SM, NM, TM). All probands and/or their parents were interviewed to determine family history, onset of hearing loss, possible causes, progression, and comorbidity, and each individual underwent physical and otologic evaluations. Audiological examinations included pure-tone audiometry, auditory steady-state response, or conditioned orientation reflex audiometry, according to the age of the patient. Severity of hearing loss was determined according to the recommendations of the Genetic Deafness study group [52]. Temporal bones and inner ears were inspected by computed tomography. Physical findings and clinical data other than audiological or otological tests were obtained by visual inspection of the entire body, palpation of the head and neck, and interview of the affected individuals or their parents. Body weight and height, motor and neuronal development, as well as behavior and cognition were checked.

### Genetic analysis

Genomic DNA samples were extracted from blood collected from the probands and their family members. Before conducting WES, probands were screened for *GJB2* or mitochondrial m.1555A>G or m.3243A>G mutations, which are frequent causes of hearing loss in Japanese patients [26].

For families 1 and 2, genomic DNA was subjected to capture of whole-exome regions, using a Nextera Rapid Capture Exome kit (Illumina) [53], and to massively parallel sequencing, using the HiSeq2500 platform (Illumina). Paired-end read sequences were checked for quality and mapped onto the human reference genome with decoy sequence (hs37d5) using BWE-mem (v0.7.5a), and variants and genotypes were called using the Genome Analysis Toolkit 3.4.46 (GATK) [54] and HaplotypeCaller, after removal of duplicated reads using Picard (v1.106). Individual variants were joint-called together with in-house data (WES data, N = 498; and whole-genome sequencing data, N = 1,037) [55] using GenotypeGVCFs. Variants and genotypes were filtered and refined using the Variant Quality Score Recalibration workflow, followed by CalculateGenotypePosteriors and VariantFiltration, using previously obtained in-house population WGS data. In the case of the proband of family 3, whole-exome regions were captured using SureSelect Human All Exon v5 (Agilent Technologies) and sequenced using the HiSeq4000 platform (Illumina). Both procedures provided a mean sequence coverage of ≥ 100×, with > 95% of target bases having ≥ 20× coverage (S1 Table).

To identify candidate pathogenic variants, changes considered to affect the encoded protein were filtered according to minor allele frequency (MAF) (≥ 0.001 for autosomal-dominant inheritance mode, ≥ 0.003 for X-linked and autosomal-recessive inheritance mode) in public databases: Database of Single Nucleotide Polymorphisms (dbSNP142) [56], 1000Genomes [57], NHLBI Exome Variant Server (ESP6500) [58], Exome Aggregation Consortium (ExAC) [42], gnomAD [28], and the Human Genetic Variation Database (HGVD) ver1.42 for Japanese subjects [59]. In addition, variants from families 1 and 2 were filtered for variants from in-house data with MAF ≥ 0.003, while those from family 3 were filtered for variants with MAF ≥ 0.01 in 600 individuals and family members with or without congenital brain anomaly or hearing loss. The threshold MAF for autosomal-dominant variants was set to 0.001, according to the criterion of “automatically benign” variants for autosomal-dominant variants adopted by the CLINGEN hearing loss expert panel [60]. The threshold MAF for autosomal-recessive variants was set to 0.003 in population databases and 0.005 in Japanese population databases, to avoid excluding an established pathogenic variant in *CDH23* (NM_022124: c.719C>T) [61, 62], which is present at an exceptionally high MAF in Japanese population databases (0.00248139 in HGVD [59] and 0.00327511 in our in-house data [55]).

The remaining variants were prioritized into three categories prior to analysis of their co-segregation with the disease: Tier 1 genes, 293 genes reported in OMIM as associated with nonsyndromic or syndromic hearing loss (S2 Table); Tier 2 genes, 328 genes associated with hearing loss in animal models by the Mouse Genome Informatics [63] or International Mouse Phenotyping Consortium [27], and not included in Tier 1 (S3 Table); and Tier 3 genes, all other genes. The effect of variants on splicing was predicted using Human Splice Finder 3.0 [24]. Candidate variants were validated by Sanger sequencing, using PrimeSTAR polymerase (TaKaRa BIO). Samples from individual I-3 in family 1 and III-2 in family 4 were subjected to targeted analysis of 154 deafness genes (S7 Table), which was modified from the previous study [11]. The analytical procedures including thresholds of MAF to filter candidate pathogenic variants were identical to those used for WES analysis. For paternity testing in family 3 and 4, the STR markers D1S80 (MCT118) and D17S5 (YNZ22) were amplified using AmpliTaqGold 360 (ThermoFisher Scientific) and analyzed using a BioAnalyzer 2100. Primers used for validation of candidate variants by Sanger sequencing and STR marker analysis are shown in S8 Table.

### Splicing and gene expression analyses of *SLC12A2* transcripts

A genomic region spanning from within intron 20 of *SLC12A2* to within intron 22 (2,507 bp) was amplified from the proband of family 2 and inserted into the vector pSpliceExpress (Addgene: 32485) [30]. Host HEK293T cells (RIKEN Bio Resource Center) were transfected with each vector using Lipofectamine 2000 (ThermoFisher Scientific) and incubated for 2 days (three independent experiments). Total RNA was extracted using the RNeasy mini kit (QIAGEN) and reverse-transcribed with SuperScript III (ThermoFisher Scientific). PCR was carried out at 35 cycles of 98°C for 10 sec, 60°C for 10 sec, and 72°C for 30 sec, followed by 72°C for 5 min, using PrimeSTAR polymerase (TaKaRa BIO).

To quantify the endogenous expression levels of human, *M*. *fascicularis*, and *M*. *musculus SLC12A2*/*Slc12a2* transcripts, specific primers to distinguish the exon 21-included transcript (variant 1 in human (NM_001046.2), variant X1 in *M*. *fascicularis* (XM_005557674.2), and variant 1 in *M*. *musculus* (NM_009194.3)) from the exon 21-skipped transcript (variant 2 in human (NM_001256461.2), variant X2 in *M*. *fascicularis* (XM_005557675.2), and variant X1 in *M*. *musculus* (XM_006525732.3)) were designed and analyzed in triplicate experiments, using a Power SybrGreen kit (Agilent Technologies) and the QuantStudio3 system (Agilent Technologies), according to the manufacturer’s protocols. All primer sets used to amplify the genomic region or transcripts of *SLC12A2*, as well as the glyceraldehyde-3-dehydrogenase (*GAPDH*) housekeeping gene, are listed in S8 Table. cDNA templates were generated from human brain total RNA (TaKaRaBIO) and total RNA extracted from *M*. *fascicularis* cochleae (a male, 3 years old, from the Primate Research Center, Ibaraki, Japan [64]), and various tissues (cerebral cortex, cerebellum, cochlea, choroid plexus, eye, spleen, liver, intestine, kidney, and ovary) from *M*. *musculus* strain FVB/NJ (both sexes except ovary, 4–12 weeks old, N = 3). RT-PCR was carried out using the Type-iT Multi Detection PCR kit (QIAGEN). The PCR conditions were as follows: 95°C for 5 min, followed by 40 cycles of 95°C for 30 sec, 54°C for 90 sec, and 72°C for 40 sec, and a final cycle of 68°C for 5 min. Statistical analysis was conducted by one-way ANOVA, combined with the Tukey-Kramer test. *P* < 0.05 was considered statistically significant.

### Cl^−^ influx assay of cells transfected with recSLC12A2 constructs

A plasmid expressing WT SLC12A2, amino-terminally attached to Cl^−^-sensitive YFP (NT13) [34], was purchased from Addgene (49060) and cloned into the vector pSBtet-Pur (Addgene: 60507) [65]. The D981Y and P988T missense mutations were introduced by site-directed mutagenesis. A recSLC12A2 construct without the exon 21-encoded region, representing the expected transcript generated due to the c.2930–2A>G mutation (Δex21), was generated by multistep PCR [66]. An mTurquoise2 (mTq2) construct, cloned into the vector pSBbi-Bla (Addgene: 60526), was also generated, and the mTq2 fluorescence was used as a reference to correct for fluctuations in the observed YFP fluorescence. Stable HEK293T cell lines were established using these constructs, as previously described [65]. Expression of recSLC12A2 and its variants was induced by the application of doxycycline (1 μg/ml) 1 day prior to experiments. mTq2 was constitutively expressed. Cells were cultured in DMEM containing 10% FBS and penicillin/streptomycin (100 U/ml). Prior to the Cl^−^ influx assay, cells were cultured in suspension for 30 min at room temperature in a low-Cl^−^ buffer, containing 135 mM sodium gluconate, 5 mM KCl, 0.5 mM CaCl_2_, 0.5 mM MgCl_2_, and 15 mM HEPES (pH 7.4). An aliquot (150 μl) of each cell suspension was transferred to a 96-well plate (approximately 1.5 × 10^5^ cells/well). The Cl^−^ influx assay was initiated by an automated injection of 100 μl of a high-Cl^−^ buffer, containing 135 mM NaCl, 5 mM KCl, 0.5 mM CaCl_2_, 0.5 mM MgCl_2_, and 15 mM HEPES (pH 7.4), in a Synergy Neo2 plate reader (BioTek). The fluorescence intensities of YFP (F_YFP_) and mTq2 (F_mTq2_) were simultaneously measured over time. The fluorescence ratio, F_YFP_/F_mTq2_, was plotted against the corresponding measurement time, and the ion transport activity (sec^−1^) was determined by exponential curve-fit analyses. All band-pass and dichroic filters and mirrors were purchased from Chroma Technologies. One-way ANOVA, combined with the Tukey-Kramer test, was performed for multiple comparisons, and *p* < 0.05 was considered statistically significant.

### Cell imaging

For fluorescence imaging of the recSLC12A2 constructs, cells were fixed in 4% formaldehyde for 5 min at room temperature. Images were captured using an A1R confocal microscope with a Plan Apo 60× oil objective (Nikon).

### Immunohistochemistry

Cochleae were dissected from saline-perfused, formalin-fixed temporal bones of young adult female *M*. *fascicularis* (3 years old, N = 3) and decalcified with 0.1 M EDTA/phosphate buffer at 4°C for at least 6 weeks, then embedded in paraffin. The 5 μm sections were rehydrated, pretreated with sodium citrate buffer, then blocked in PBS containing 5% FBS, 1% BSA, and 0.05% Tween-20. The primary antibodies used in this study were rabbit antiserum against SLC12A2, generated using the amino-terminal region of the protein as an immunogen, and thus able to detect both the exon 21-included and -skipped isoforms (Abcam: ab59791, 1:100), mouse monoclonal antibody against ATP1B1 (Santa Cruz Biotechnology: sc21713, 1:400), and goat antiserum against KCNQ1 (Santa Cruz Biotechnology: sc10646, 1:200). Sections were incubated with primary antibodies at 4°C overnight, and signals were visualized using appropriate secondary antibodies, conjugated with either Alexa 488 or 568, followed by counterstaining with DAPI. Images were captured by fluorescence (DM2500, Leica Microsystems) and confocal (Axio 700) microscopy.

## Supporting information

S1 Fig Audiograms and estimated audiograms for family members in this study. Estimated hearing levels, measured by auditory steady-state response (A, B, H), and audiograms measured by conditioned orientation reflex audiometry (C) or pure-tone audiometry (D–G) are shown. Open circle: right ear, air conduction; X: left ear, air conduction; open triangle: bilateral ears, air conduction; [: right ear, bone conduction;]: left ear, bone conduction. Right or left downward arrows indicate undetectable levels of left or right ears with the corresponding sound levels at respective frequencies.
(PDF)

S2 Fig Partial electropherograms generated by Sanger sequencing. Results of sequencing of the exon 21 region in the parents of each proband to validate co-segregation of the respective *SLC12A2* variants with the phenotypes (family 1, A) or *de novo* variants (families 2 and 3, B and C).
(PDF)

S3 Fig Parenthood testing in family 2 and 3. (A) In family 2, 217 protein-affecting (non-synonymous, stop gain, indel, and splicing) variants with MAF < 0.003 detected in proband (III-2) were sub-classified and shown. Among 21 variants detected only in the proband, 15 of them were with low quality scores or on repeated elements and are shown in parenthesis. (B) In family 3, STR markers D1S80 (16 bp repeats) and D17S30 (70 bp repeats) in the proband and his parents. The image was generated using a BioAnalyzer 2100. M, 100 bp ladder markers.
(PDF)

S4 Fig Non-candidate variants in family 3. (A,B) Results of analysis of *TECTA* (A) and *ACAN* (B) variants in the proband (III-2) and his parents (II-3 and II-4) with normal hearing.
(PDF)

S5 Fig *SLC12A2* variant found in III-2 of family 4. (A) Pedigree of family 4. (B,C) Audiograms of the proband measured by conditioned orientation reflex audiometry (B) or estimated by auditory steady-state response (C). (D) Details of the *SLC12A2* variant. (E-G) Partial electropherograms of the exon 21 region generated by Sanger sequencing in proband (E) and the parents (F,G). (H). Parenthood testing in family 4.
(PDF)

S6 Fig Partial results of multiple alignment of eight SLC12A family proteins. Amino acid sequences were aligned using a constraint-based multiple alignment tool (NCBI, https://www.ncbi.nlm.nih.gov/tools/cobalt/cobalt.cgi). Numbers indicate the positions of amino acid residues at each end. Red, identical residues among the eight proteins; blue, identical residues among at least three proteins without a gap. Residues encoded by exon 21 of *SLC12A2* are underlined. The other SLC12A family protein, SLC12A8 (NP_078904), was excluded from the analysis because its amino acid sequence was not sufficiently similar to those of the other eight proteins in this region. SLC12A1, NP_000329; SLC12A2, NP_001037; SLC12A3, NP_000330; SLC12A4, NP_005063; SLC12A5, NP_001128243; SLC12A6, NP_598408; SLC12A7, NP_006589; SLC12A9, NP_064631.
(PDF)

S7 Fig Expression levels of *Slc12a2* transcripts determined by qRT-PCR. (A) Expression levels of each *Slc12a2* transcript and their ratio in the tissues shown in Fig 3C and 3D. Data are from triplicate analyses of *M*. *musculus* tissue samples (n = 3), a human brain, and the left or right whole cochlea from *M*. *fascicularis*. (B) *P* values for the differential expression levels of the exon 21-included (*top right*) or -skipped (*bottom left*) transcripts between two tissues. One-way ANOVA, Tukey-Kramer multiple comparison test. Columns highlighted in orange or pale orange indicate that *p* values were < 0.005 or < 0.05, respectively.
(PDF)

S8 Fig Assessment of *Slc12a2* transcripts in mammalian tissues by RT-PCR. (A) Forty cycles of RT-PCR were used to detect exon 21-included or -skipped transcript variants of *Slc12a2* in *M*. *musculus* tissues. The longer PCR product (219 bp, filled arrowhead) and the shorter products (171 bp, open arrowhead) in the cortex and cerebellum were confirmed to be the exon 21-included and -skipped transcripts, respectively, by extraction of each product from gels following electrophoresis and Sanger sequencing. PCR band of the whole cochlea was also confirmed to be the exon 21-included transcript by direct sequencing. (B) Forty cycles of RT-PCR to detect exon 21-included or -skipped transcript variants of *SLC12A2* in a *H*. *sapiens* brain and a left whole cochlea from *M*. *fascicularis*. The size of each transcript is indicated by an open or filled arrowhead. M, 100 bp ladder marker.
(PDF)

S9 Fig Summary of *SLC12A2* expression in mammalian tissues. (A) Summary of qRT-PCR data presented in Fig 3C and 3D and S7 Fig. The number of “+” symbols roughly reflects the magnitude of the expression levels of each transcript in the indicated tissues. (B) Summary of RT-PCR data presented in S8 Fig. Detection of marginal levels of the transcript is indicated by “±”.
(PDF)

S10 Fig Junction expression of *SLC12A2* in human tissues. Data are derived from the GTEx Portal [30]. Blue arrow, tissues in which exon 21 skipping was detected by RNA-seq. The darker the red color, the more intense the observed exon 21 skipping.
(PDF)

S11 Fig Histology of *M*. *fascicularis* cochlear tissues. (A) Paraffin sections of adult *M*. *fascicularis* cochleae were stained with hematoxylin and eosin. Areas of the organ of Corti (oc), spiral limbus (slm), spiral prominence (sp), and stria vascularis (stv) are shown with area bars; rm, Reissner’s membrane; slg, spiral ligament; sm, scala media; sv, scala vestibuli. (B,C) Images of the cochlear lateral walls. Cochlear specimens were incubated with rabbit antiserum against SLC12A2 (green) and goat antiserum against KCNQ1 (red) and counterstained with DAPI (blue) (B) or treated without primary antibodies (C). Scale bar, 50 μm.
(TIF)

S1 Table Summary of whole-exome sequencing in this study. (PDF)

S2 Table List of genes categorized in Tier 1 in this study. (PDF)

S3 Table List of genes categorized in Tier 2 in this study. (PDF)

S4 Table Primary candidate variants co-segregated with hearing loss in family 1. (PDF)

S5 Table Primary candidate variants found in family 2 with hearing loss. (PDF)

S6 Table Primary candidate variants found in family 3 with hearing loss. (PDF)

S7 Table List of deafness genes for targeted sequencing. (PDF)

S8 Table Primers used in this study. (PDF)
