# Supplementary figures and images for: Variants encoding a restricted carboxy-terminal domain of SLC12A2 cause hereditary hearing loss in humans
Source: PLoS Genet. 2020 Apr 15;16(4):e1008643. doi: 10.1371/journal.pgen.1008643 (PMC7159186; doi:10.1371/journal.pgen.1008643)

A

Family 1, III-1

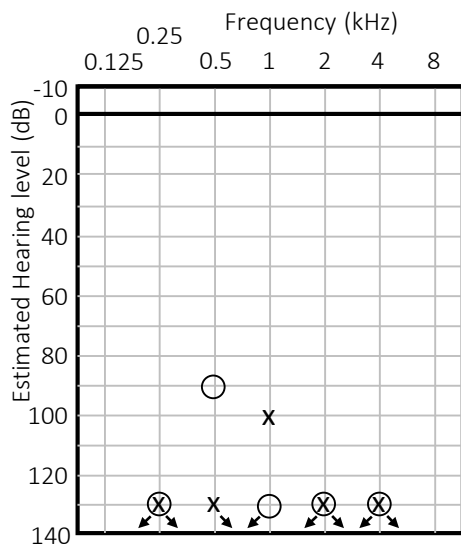

B

Family 1, III-2

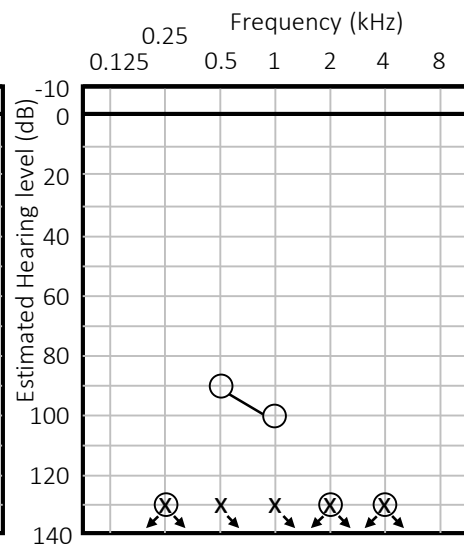

C

Family 1, III-3

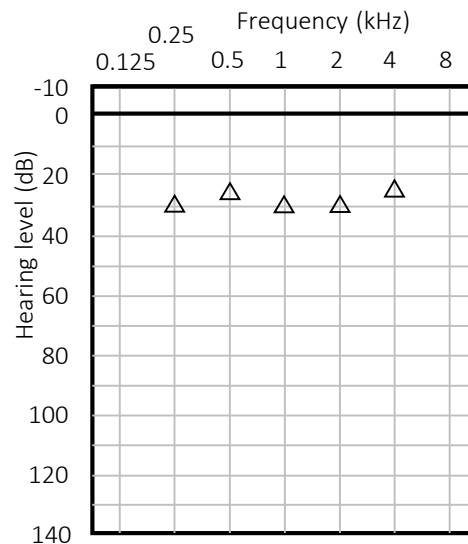

D

Family 1, II-3

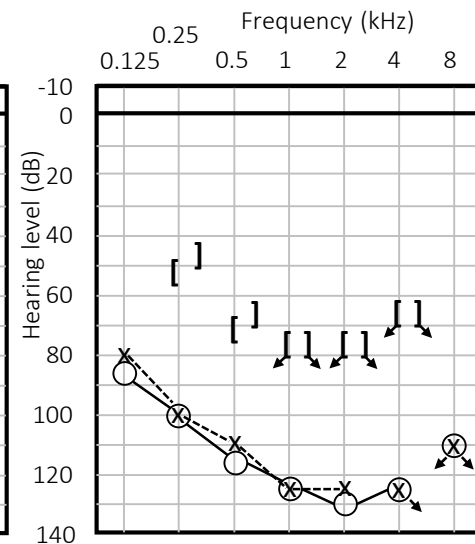

E

Family 1, II-4

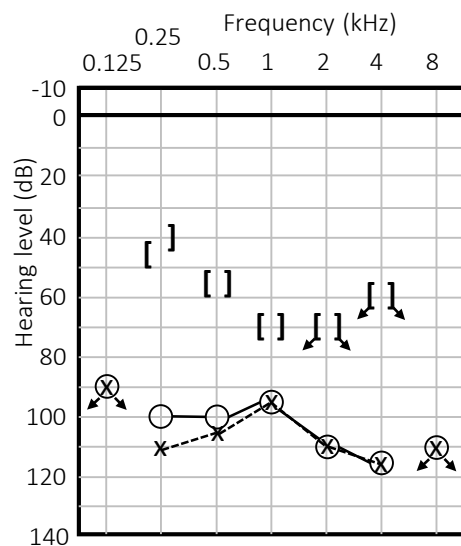

F

Family 1, I-3

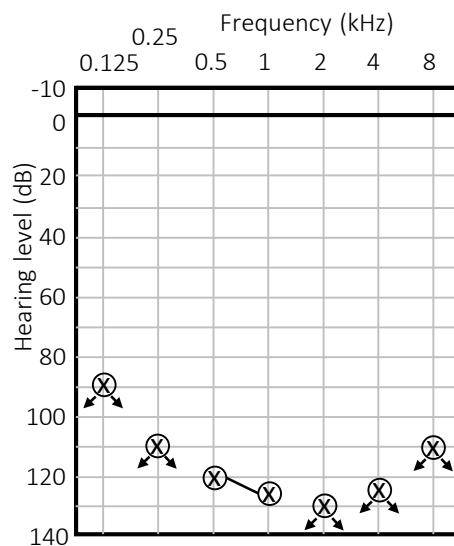

G

Family 1, I-4

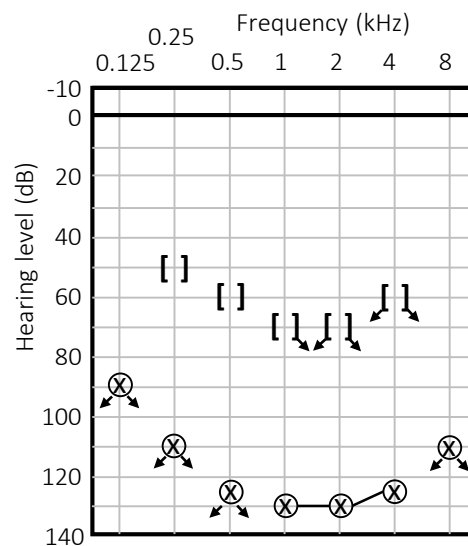

H

Family 3, III-2

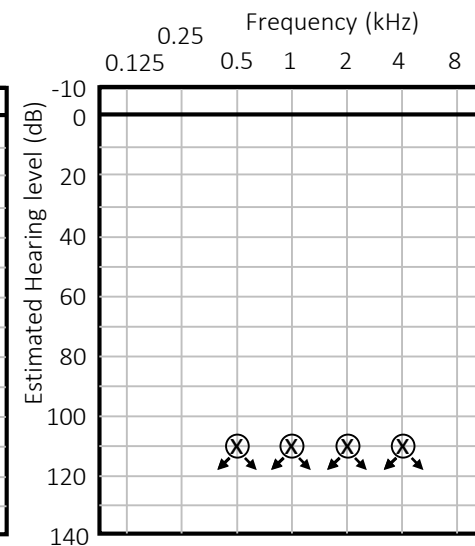

Supplement: S1 Fig — Estimated hearing levels, measured by auditory steady-state response (A, B, H), and audiograms measured by conditioned orientation reflex audiometry (C) or pure-tone audiometry (D–G) are shown. Open circle: right ear, air conduction; X: left ear, air conduction; open triangle: bilateral ears, air conduction; [: right ear, bone conduction;]: left ear, bone conduction. Right or left downward arrows indicate undetectable levels of left or right ears with the corresponding sound levels at respective frequencies. (PDF) [file pgen.1008643.s001.pdf]

A

Family 1, II-1

Family 1, II-4

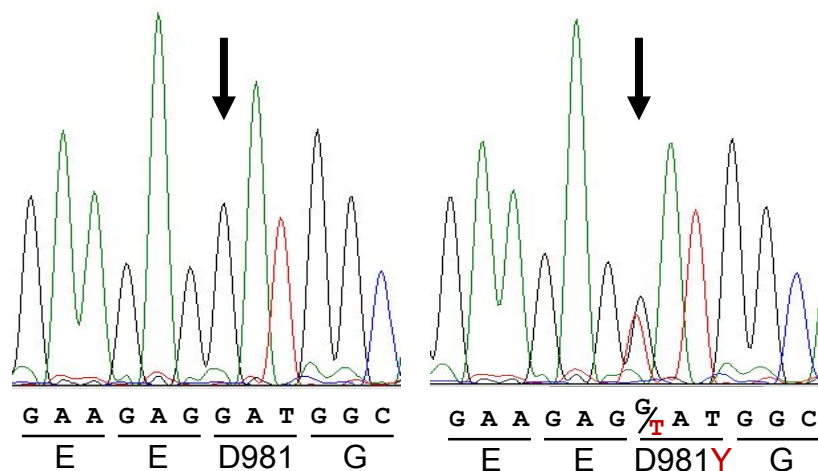

B

Family 2, II-3

Family 2, II-4

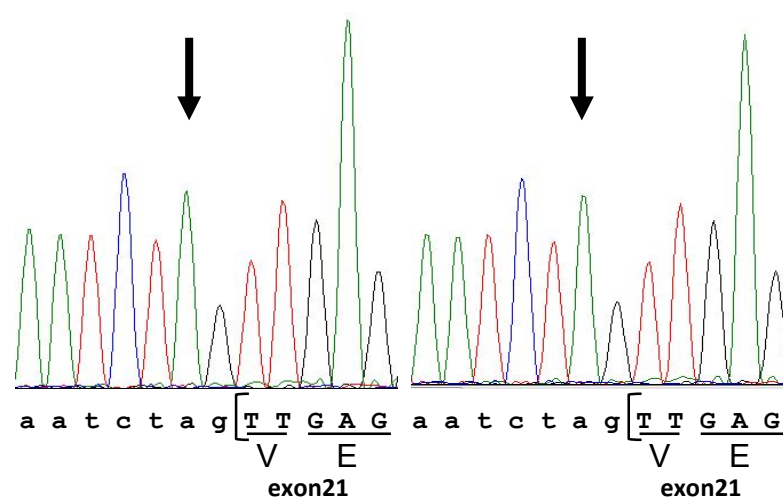

C

Family 3, II-3

Family 3, II-4

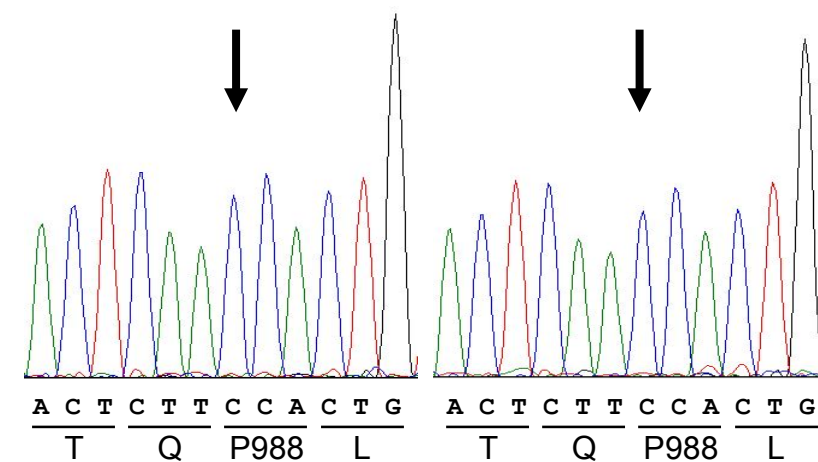

Supplement: S2 Fig — Results of sequencing of the exon 21 region in the parents of each proband to validate co-segregation of the respective SLC12A2 variants with the phenotypes (family 1, A) or de novo variants (families 2 and 3, B and C). (PDF) [file pgen.1008643.s002.pdf]

A

Family 2

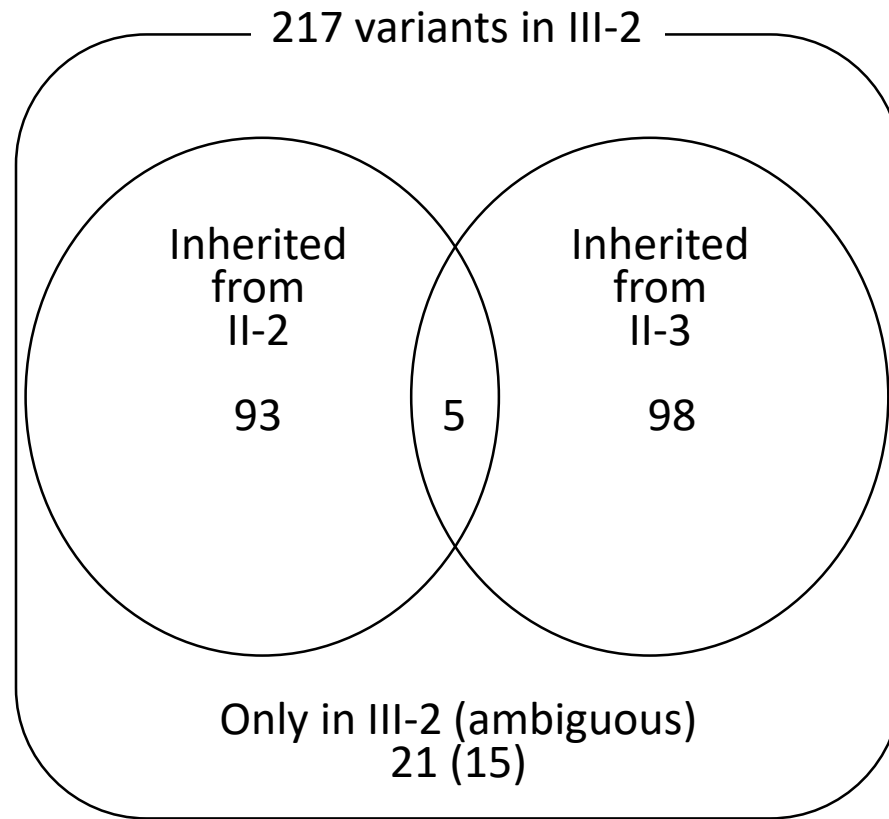

B

Family 3

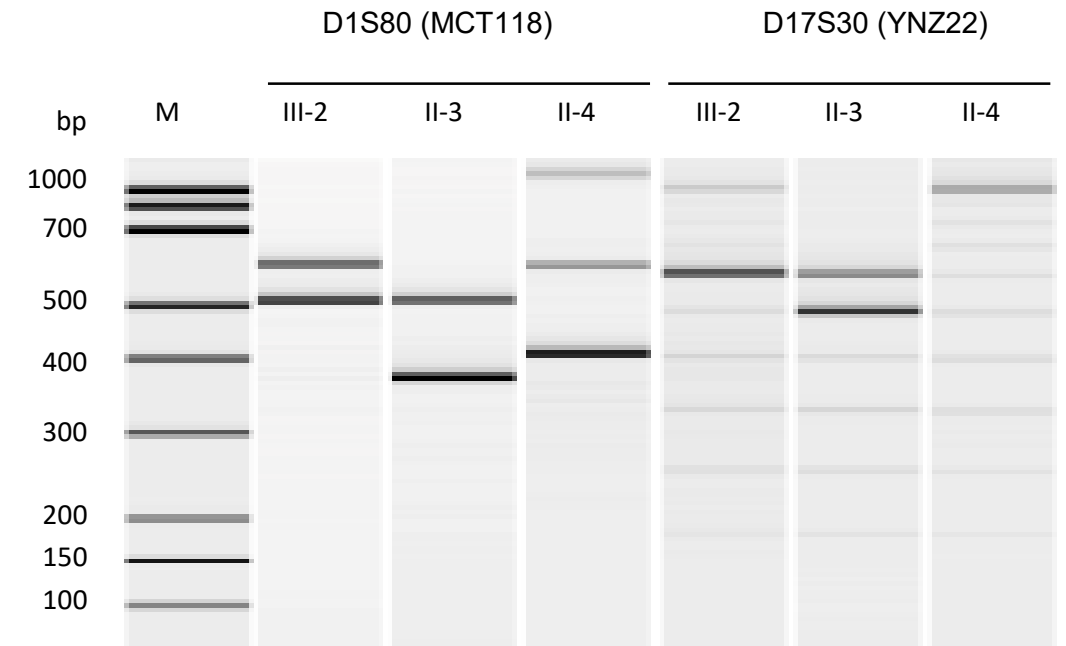

Supplement: S3 Fig — (A) In family 2, 217 protein-affecting (non-synonymous, stop gain, indel, and splicing) variants with MAF < 0.003 detected in proband (III-2) were sub-classified and shown. Among 21 variants detected only in the proband, 15 of them were with low quality scores or on repeated elements and are shown in parenthesis. (B) In family 3, STR markers D1S80 (16 bp repeats) and D17S30 (70 bp repeats) in the proband and his parents. The image was generated using a BioAnalyzer 2100. M, 100 bp ladder markers. (PDF) [file pgen.1008643.s003.pdf]

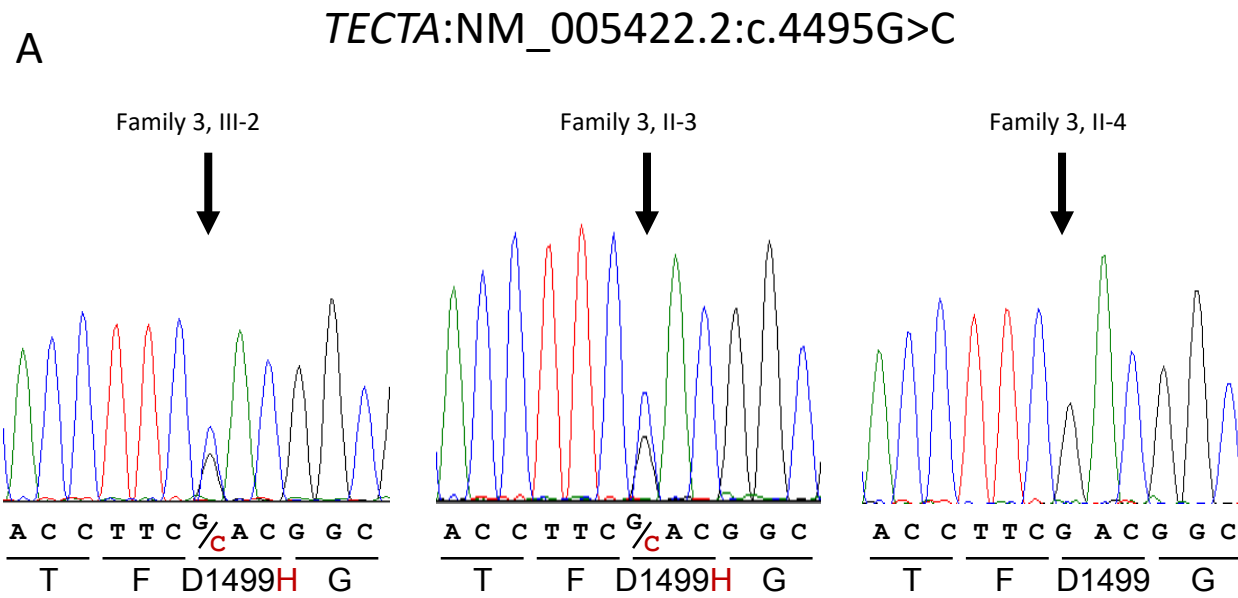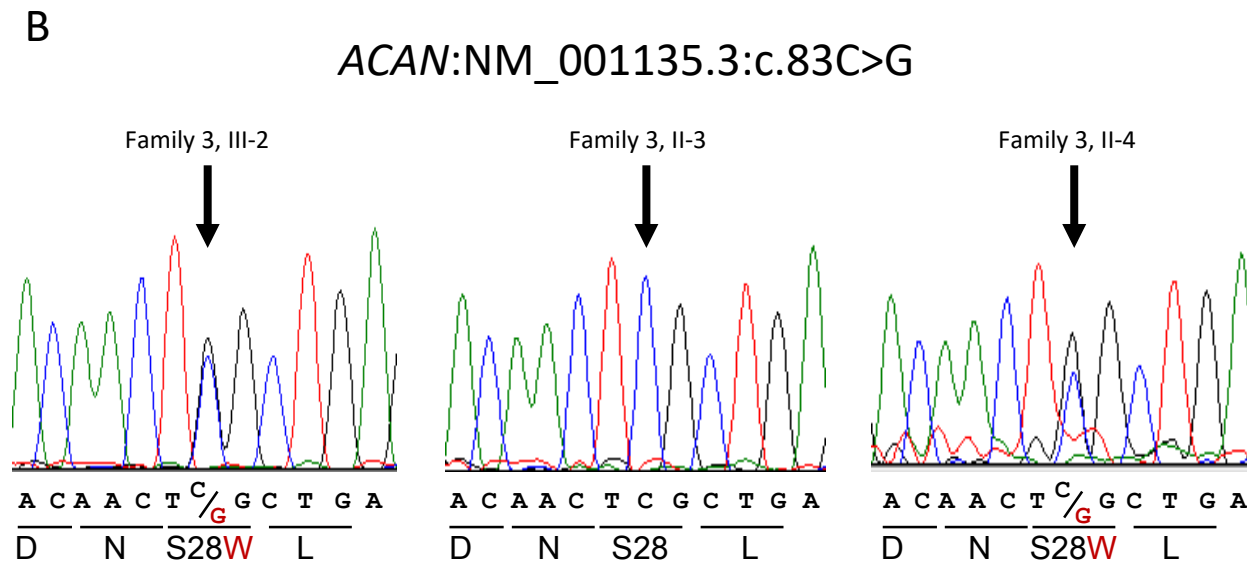

Supplement: S4 Fig — (A,B) Results of analysis of TECTA (A) and ACAN (B) variants in the proband (III-2) and his parents (II-3 and II-4) with normal hearing. (PDF) [file pgen.1008643.s004.pdf]

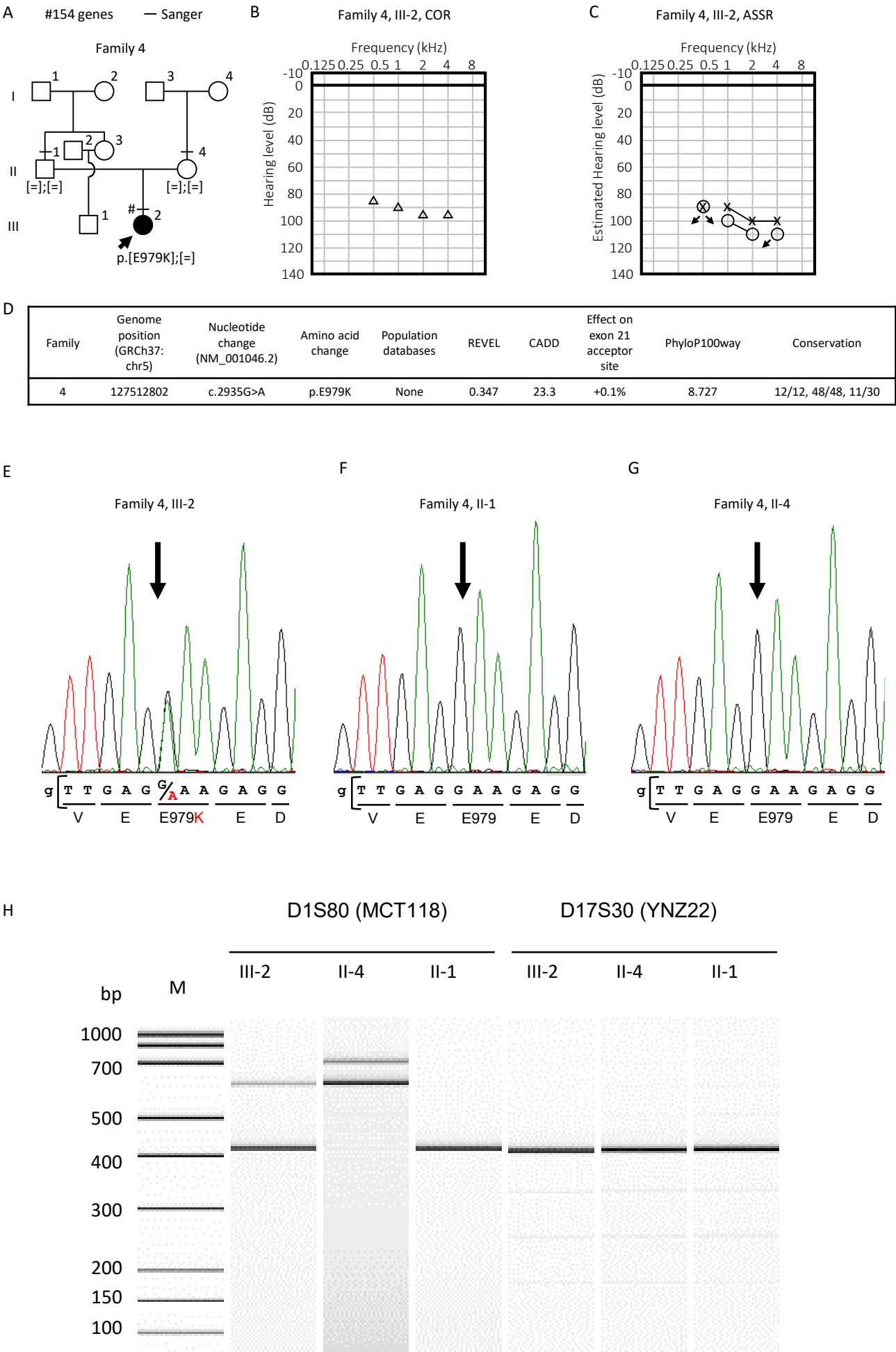

Supplement: S5 Fig — (A) Pedigree of family 4. (B,C) Audiograms of the proband measured by conditioned orientation reflex audiometry (B) or estimated by auditory steady-state response (C). (D) Details of the SLC12A2 variant. (E-G) Partial electropherograms of the exon 21 region generated by Sanger sequencing in proband (E) and the parents (F,G). (H). Parenthood testing in family 4. (PDF) [file pgen.1008643.s005.pdf]

A *M. musculus* tissues

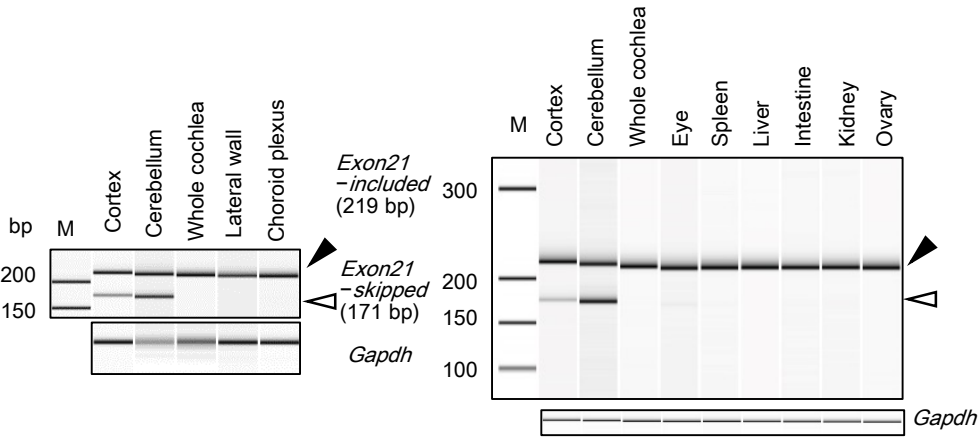

B Primate tissues

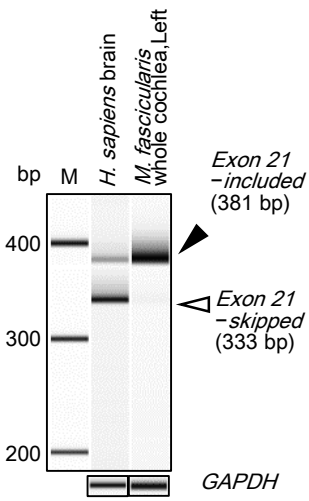

Supplement: S8 Fig — (A) Forty cycles of RT-PCR were used to detect exon 21-included or -skipped transcript variants of Slc12a2 in M. musculus tissues. The longer PCR product (219 bp, filled arrowhead) and the shorter products (171 bp, open arrowhead) in the cortex and cerebellum were confirmed to be the exon 21-included and -skipped transcripts, respectively, by extraction of each product from gels following electrophoresis and Sanger sequencing. PCR band of the whole cochlea was also confirmed to be the exon 21-included transcript by direct sequencing. (B) Forty cycles of RT-PCR to detect exon 21-included or -skipped transcript variants of SLC12A2 in a H. sapiens brain and a left whole cochlea from M. fascicularis. The size of each transcript is indicated by an open or filled arrowhead. M, 100 bp ladder marker. (PDF) [file pgen.1008643.s008.pdf]

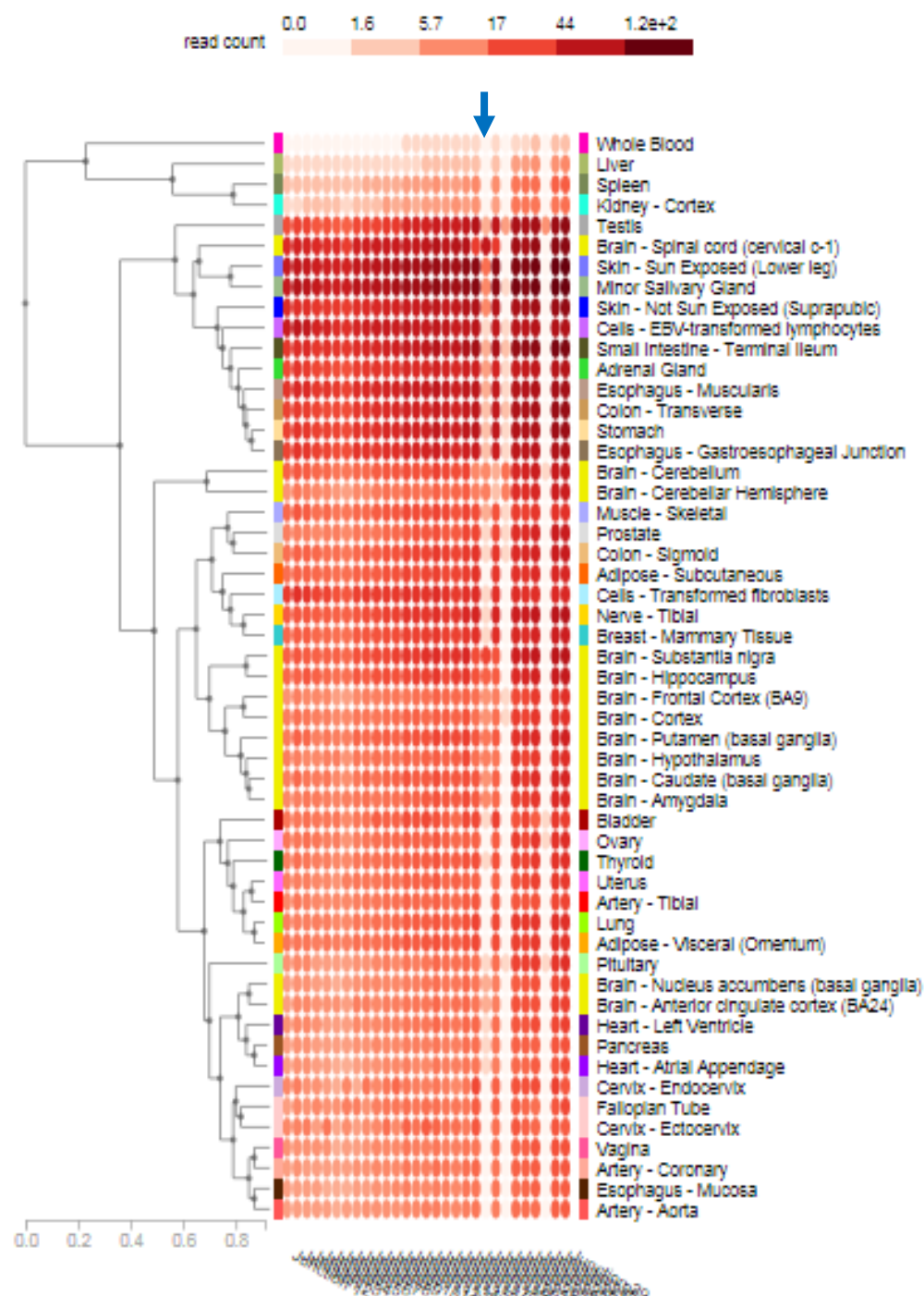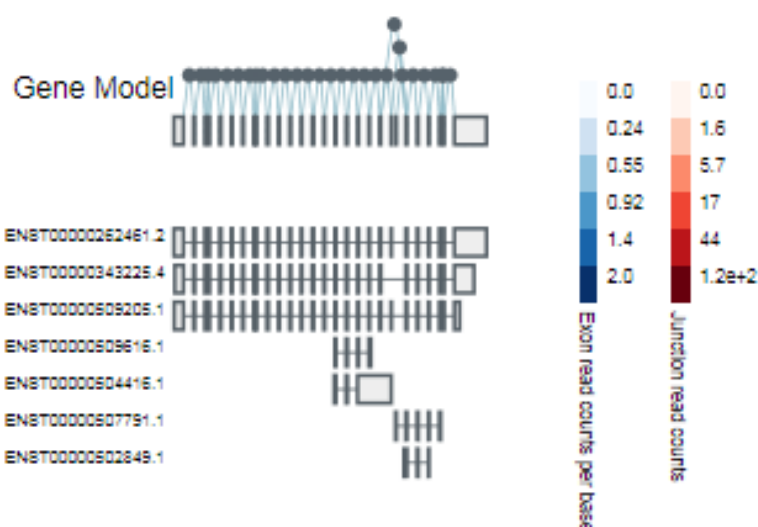

Supplement: S10 Fig — Data are derived from the GTEx Portal [30]. Blue arrow, tissues in which exon 21 skipping was detected by RNA-seq. The darker the red color, the more intense the observed exon 21 skipping. (PDF) [file pgen.1008643.s010.pdf]

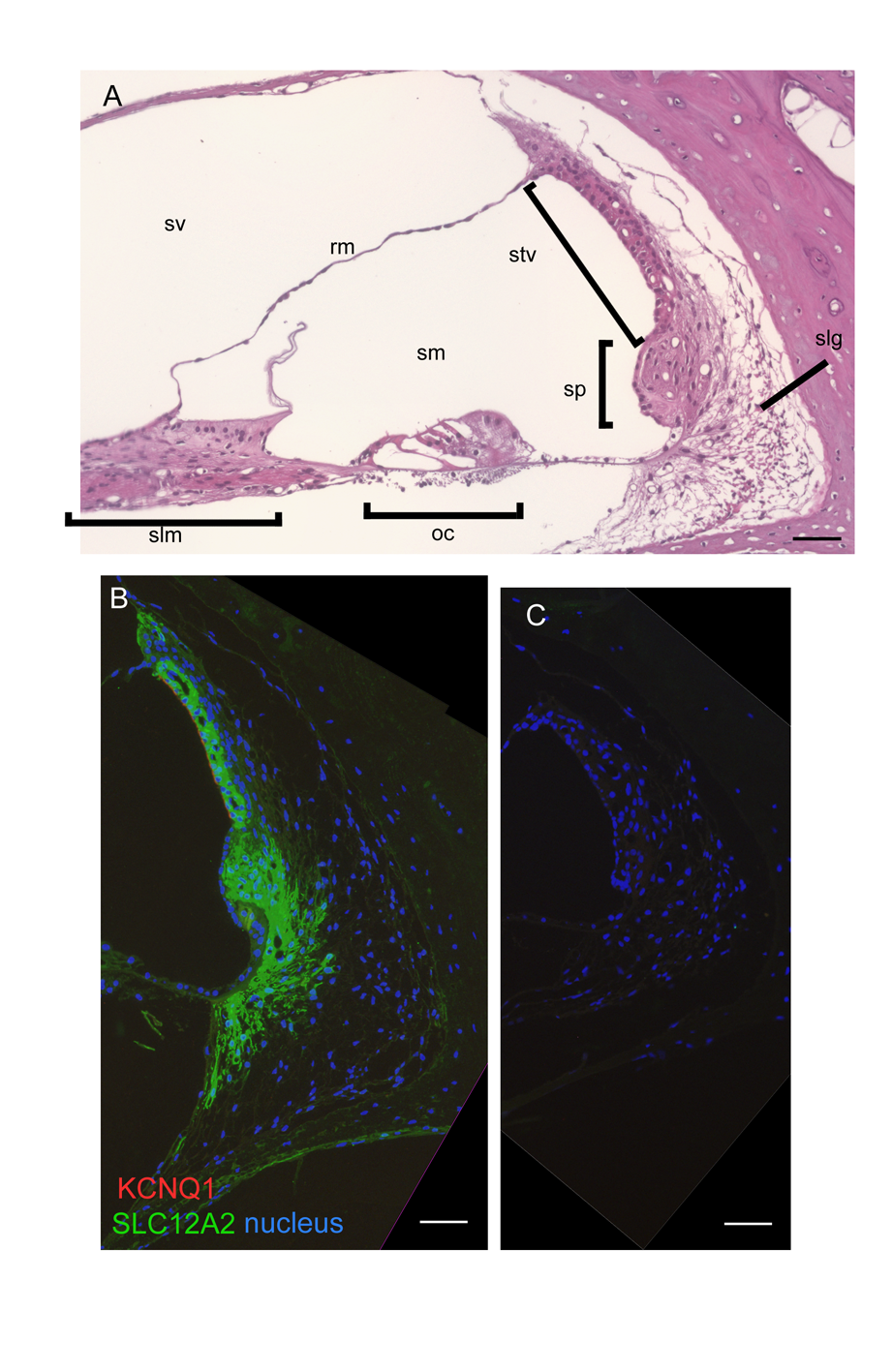

Supplement: S11 Fig — (A) Paraffin sections of adult M. fascicularis cochleae were stained with hematoxylin and eosin. Areas of the organ of Corti (oc), spiral limbus (slm), spiral prominence (sp), and stria vascularis (stv) are shown with area bars; rm, Reissner’s membrane; slg, spiral ligament; sm, scala media; sv, scala vestibuli. (B,C) Images of the cochlear lateral walls. Cochlear specimens were incubated with rabbit antiserum against SLC12A2 (green) and goat antiserum against KCNQ1 (red) and counterstained with DAPI (blue) (B) or treated without primary antibodies (C). Scale bar, 50 μm. (TIF) [file pgen.1008643.s011.tif]
