# Supplementary material for: Variants encoding a restricted carboxy-terminal domain of SLC12A2 cause hereditary hearing loss in humans
Source: PLoS Genet. 2020 Apr 15;16(4):e1008643. doi: 10.1371/journal.pgen.1008643 (PMC7159186; doi:10.1371/journal.pgen.1008643)
Supplement: S6 Fig — Amino acid sequences were aligned using a constraint-based multiple alignment tool (NCBI, https://www.ncbi.nlm.nih.gov/tools/cobalt/cobalt.cgi). Numbers indicate the positions of amino acid residues at each end. Red, identical residues among the eight proteins; blue, identical residues among at least three proteins without a gap. Residues encoded by exon 21 of SLC12A2 are underlined. The other SLC12A family protein, SLC12A8 (NP_078904), was excluded from the analysis because its amino acid sequence was not sufficiently similar to those of the other eight proteins in this region. SLC12A1, NP_000329; SLC12A2, NP_001037; SLC12A3, NP_000330; SLC12A4, NP_005063; SLC12A5, NP_001128243; SLC12A6, NP_598408; SLC12A7, NP_006589; SLC12A9, NP_064631. (PDF) [file pgen.1008643.s006.pdf]

```
SLC12A1 852 EESGGIRGLFKKAGKLNITKTTPKKDGSINTSQSMH-------- 887
SLC12A2 958 KKSD--LDTSKPLSEKPITHKVEEEDGKTATQPLLKKESKGPIV 994
SLC12A3 801 KEASA-------------RGARPSVSGALDPKALVKEE------ 825
SLC12A4 839 ERYLE--------------------------------------- 843
SLC12A5 842 ERFSE--------------------------------------- 846
SLC12A6 904 EQFSE--------------------------------------- 908
SLC12A7 839 ERFGG--------------------------------------- 843
SLC12A9 685 ATVAD-------ALKMNKNVVLARASGALPPERLSRGSGG---- 717

888 -VGEFNQKLVEASTQFKKKQEKGTIDVWWLFDDGGLTLLIPYILTLRKKWKDCKLRIYV 945
995 PLNVADQKLLEASTQFQKKQGKNTIDVWWLFDDGGLTLLIPYLLTTKKKWKDCKIRVFI 1059
826 ----------QATTIFQSEQGKKTIDIYWLFDDGGLTLLIPYLLGRKRRWSKCKIRVFV 874
844 ----------------------GHIDVWWIVHDGGMLMLLPFLLRQHKVWRKCRMRIFT 880
847 ----------------------GSIDVWWIVHDGGMLMLLPFLLRHHKVWRKCKMRIFT 883
909 ----------------------GNIDVWWIVHDGGMLMLLPFLLKQHKVWRKCSIRIFT 945
844 ----------------------GHIDVWWIVHDGGMLMLLPFLLRQHKVWRKCRMRIFT 880
718 -TSQLHHVDVWPLNLLRPRGGPGYVDVCGLF-----LLQMATILGMVPAWHSARLRIFL 770
```
