# Supplementary material for: Variants encoding a restricted carboxy-terminal domain of SLC12A2 cause hereditary hearing loss in humans
Source: PLoS Genet. 2020 Apr 15;16(4):e1008643. doi: 10.1371/journal.pgen.1008643 (PMC7159186; doi:10.1371/journal.pgen.1008643)
Supplement: S7 Fig — (A) Expression levels of each Slc12a2 transcript and their ratio in the tissues shown in Fig 3C and 3D. Data are from triplicate analyses of M. musculus tissue samples (n = 3), a human brain, and the left or right whole cochlea from M. fascicularis. (B) P values for the differential expression levels of the exon 21-included (top right) or -skipped (bottom left) transcripts between two tissues. One-way ANOVA, Tukey-Kramer multiple comparison test. Columns highlighted in orange or pale orange indicate that p values were < 0.005 or < 0.05, respectively. (PDF) [file pgen.1008643.s007.pdf]

A

Species	Tissue	*Slc12a2*, exon 21-included/ *Gapdh*	*Slc12a2*, exon 21-skipped/ *Gapdh*	Ratio, exon 21-included/skipped
M. musculus	Cortex	$8.350 \times 10^{-3} \pm 3.116 \times 10^{-3}$	$3.851 \times 10^{-3} \pm 1.624 \times 10^{-3}$	$2.197 \pm 2.050 \times 10^{-2}$
M. musculus	Cerebellum	$1.402 \times 10^{-2} \pm 1.257 \times 10^{-4}$	$1.963 \times 10^{-2} \pm 9.723 \times 10^{-4}$	$7.152 \times 10^{-1} \pm 8.099 \times 10^{-4}$
M. musculus	Whole cochlea	$3.099 \times 10^{-1} \pm 3.694 \times 10^{-2}$	$1.194 \times 10^{-2} \pm 1.873 \times 10^{-3}$	$2.606 \times 10 \pm 1.343$
M. musculus	Lateral wall	$2.963 \times 10^{-1} \pm 6.741 \times 10^{-2}$	$6.050 \times 10^{-3} \pm 1.691 \times 10^{-3}$	$3.791 \times 10 \pm 5.005 \times 10$
M. musculus	Choroid plexus	$1.624 \times 10^{-1} \pm 7.405 \times 10^{-4}$	$1.039 \times 10^{-2} \pm 1.696 \times 10^{-3}$	$1.595 \times 10 \pm 8.062$
H. sapiens	Brain	$6.680 \times 10^{-2} \pm 4.700 \times 10^{-3}$	$1.580 \times 10^{-1} \pm 3.205 \times 10^{-3}$	$4.273 \times 10^{-1} \pm 5.624 \times 10^{-2}$
M. fascicularis	Whole cochlea, left	$3.773 \times 10^{-1} \pm 1.709 \times 10^{-2}$	$5.145 \times 10^{-2} \pm 9.872 \times 10^{-4}$	$7.335 \pm 1.672 \times 10^{-1}$
M. fascicularis	Whole cochlea, right	$3.911 \times 10^{-1} \pm 3.056 \times 10^{-2}$	$5.018 \times 10^{-2} \pm 1.213 \times 10^{-3}$	7.844 ± 1.057

B

p value (Tukey–Kramer)	Cortex	Cerebelllum	Whole cochlea	Lateral wall	Choroid plexus
Cortex		0.9995625	0.0000064	0.0000098	0.001928
Cerebellum	0.0000021		0.0000076	0.0000118	0.0025474
Whole cochlea	0.0007567	0.0011236		0.9869709	0.0026617
Lateral wall	0.4872431	0.0000086	0.0077166		0.0053234
Choroid plexus	0.0038087	0.0002536	0.7559682	0.0477535	

Exon 21 -included transcript

Exon 21-skipped transcript
