# Supplementary material for: Variants encoding a restricted carboxy-terminal domain of SLC12A2 cause hereditary hearing loss in humans
Source: PLoS Genet. 2020 Apr 15;16(4):e1008643. doi: 10.1371/journal.pgen.1008643 (PMC7159186; doi:10.1371/journal.pgen.1008643)
Supplement: S9 Fig — (A) Summary of qRT-PCR data presented in Fig 3C and 3D and S7 Fig. The number of “+” symbols roughly reflects the magnitude of the expression levels of each transcript in the indicated tissues. (B) Summary of RT-PCR data presented in S8 Fig. Detection of marginal levels of the transcript is indicated by “±”. (PDF) [file pgen.1008643.s009.pdf]

A

qRT-PCR

	M. musculus					*H. sapiens*	*M. fascicularis*
	Cortex	Cerebellum	Whole cochlea	Lateral wall	Choroid plexus	Brain	Whole cochlea
Exon 21–included transcript	+	++	+++	+++	+++	+	+++
Exon 21–skipped transcript	+	++	++	+	++	++	++

B

RT-PCR

	M. musculus											*H. sapiens*	*M. fascicularis*
	Cortex	Cerebellum	Whole cochea	Lateral wall	Choroid plexus	Eye	Spleen	Liver	Intestine	Kidney	Ovary	Brain	Whole cochlea
Exon 21–included transcript	++	++	++	++	++	++	++	++	++	++	++	+	++
Exon 21–skipped transcript	++	++	-	-	-	±	-	-	-	-	-	++	±
