# Supplementary material for: Variants encoding a restricted carboxy-terminal domain of SLC12A2 cause hereditary hearing loss in humans
Source: PLoS Genet. 2020 Apr 15;16(4):e1008643. doi: 10.1371/journal.pgen.1008643 (PMC7159186; doi:10.1371/journal.pgen.1008643)
Supplement: S1 Table — (PDF) [file pgen.1008643.s012.pdf]

S1 Table. Summary of whole exome sequencing in this study.

Summary	Family 1 III-1	Family 1 II-1	Family 1 II-4	Family 2 III-2	Family 2 II-3	Family 2 II-4	Family 3 III-2
Total reads	146,797,358	149,196,713	145,011,463	159,791,988	99,789,451	100,820,483	50,386,798
Uniquely mapped reads (#)	146,608,800	149,042,607	144,847,541	159,687,493	99,696,970	100,755,750	50,386,798
Reads in targeted regions (%)	45.65	46.75	47.91	53.12	55.61	54.42	94.02
Reads in targeted regions +/—100 bp(%)	52.94	54.12	56.13	59.73	63.85	62.03	99.22
Average coverage (fold)	152.9	159.69	159.74	193.58	128.07	126.23	110.25
Median coverage (fold)	126	131	130	164	107	106	96
Targeted bases with minimum 20× coverage (%)	96.62	97.52	97.31	98.45	97.58	97.35	96.81
Variants predicted to affect amino acid residues	13265	13421	13364	13285	13243	13428	12062
