# Supplementary material for: Variants encoding a restricted carboxy-terminal domain of SLC12A2 cause hereditary hearing loss in humans
Source: PLoS Genet. 2020 Apr 15;16(4):e1008643. doi: 10.1371/journal.pgen.1008643 (PMC7159186; doi:10.1371/journal.pgen.1008643)
Supplement: S2 Table — (PDF) [file pgen.1008643.s013.pdf]

S2 Table. List of genes categorized in Tier 1 in this study.

AARS	*COL9A1*	*GIPC3*	*LRP2*	*PEX13*	*SNAI2*
ABCC8	*COL9A2*	*GJA1*	*LRTOMT*	*PEX14*	*SOST*
ABHD12	*COL9A3*	*GJB1*	*MAF*	*PEX19*	*SOX10*
ACTB	*COX10*	*GJB2*	*MAN2B1*	*PEX2*	*SOX2*
ACTG1	*CRYM*	*GJB3*	*MANBA*	*PEX26*	*SPTLC1*
ADCY1	*DCAF17*	*GJB4*	*MARVELD2*	*PEX3*	*STRC*
ADGRV1	*DCDC2*	*GJB5*	*MATP*	*PEX5*	*SUMF1*
AIFM1	*DFNA5*	*GJB6*	*MCM2*	*PEX7*	*SURF1*
ALMS1	*DFNB31*	*GLA*	*MED12*	*PHYH*	*SYNE4*
ALR	*DFNB59*	*GLB1*	*MEIS2*	*PLOD3*	*TANGO2*
ANKH	*DIABLO*	*GPSM2*	*MET*	*PMP22*	*TBC1D24*
AP1S1	*DIAPH1*	*GRHL2*	*MFN2*	*PNPT1*	*TBL1X*
ARSB	*DIAPH3*	*GRIP1*	*MIR96*	*POLD1*	*TCF21*
ATP1A3	*DLX5*	*GRXCR1*	*MITF*	*POLG*	*TCIRG1*
ATP2B2	*DNAJC3*	*GRXCR2*	*MLL2*	*POR*	*TCOF1*
ATP6B1	*DNMT1*	*GUSB*	*MORC2*	*POU3F4*	*TECTA*
ATP6V0A4	*DSPP*	*HARS*	*MPZ*	*POU4F3*	*TIMM8A*
ATP6V1B1	*DTNA*	*HARS2*	*MSRB3*	*PRDM13*	*TJP2*
ATP6V1B2	*EBR3*	*HEXA*	*MTO1*	*PRPS1*	*TK2*
ATRX	*ECE1*	*HGF*	*MYH14*	*PRRX1*	*TMC1*
BCAP31	*EDA*	*HNF1B*	*MYH9*	*PRX*	*TMIE*
BCS1L	*EDN3*	*HOMER2*	*MYO15A*	*PTPN11*	*TMPRSS3*
BDP1	*EDNRA*	*HOXA1*	*MYO1A*	*PTPRQ*	*TMPRSS5*
BSND	*EDNRB*	*HOXA2*	*MYO3A*	*RAB23*	*TNC*
BTD	*ELMOD3*	*HPS6*	*MYO6*	*RDX*	*TP63*
C10ORF2	*EPS8*	*HSD17B4*	*MYO7A*	*RPGR*	*TPMT*
CABP2	*ERCC2*	*HSPA1L*	*NAGLU*	*RRM2B*	*TPRN*
CACNA1D	*ERCC3*	*HSPA2*	*NARS2*	*SALL1*	*TRIOBP*
CATSPER2	*ERCC6*	*IARS2*	*NDP*	*SALL4*	*TRMU*
CCDC50	*ERCC8*	*IDS*	*NDRG1*	*SANS*	*TRPV4*
CD151	*ESPN*	*IDUA*	*NEFL*	*SBF2*	*TSPEAR*
CD164	*ESRRB*	*IGF1*	*NEU1*	*SCAX3*	*TTC8*
CDH23	*EYA1*	*ILDR1*	*NF1*	*SDHD*	*TWINK*
CEACAM16	*EYA4*	*ITM2B*	*NF2*	*SEMA3E*	*TWIST1*
CHD7	*FAM136A*	*KARS*	*NLRP3*	*SERAC1*	*TYR*
CIB2	*FAM65B*	*KCNE1*	*NOG*	*SERPINB6*	*UBR1*
CLCNKA	*FGF3*	*KCNJ10*	*NOTCH2*	*SF3B4*	*USH1C*
CLCNKB	*FGFR2*	*KCNJ11*	*OPA1*	*SH3TC2*	*USH2A*
CLDN14	*FGFR3*	*KCNQ1*	*OSBPL2*	*SIX1*	*UTX*
CLIC5	*FKBP14*	*KCNQ4*	*OTOA*	*SIX5*	*WFS1*
CLPP	*FLNA*	*KDM6A*	*OTOF*	*SLC17A8*	*WHRN*
CLRN1	*FOXC1*	*KIAA1199*	*OTOG*	*SLC19A2*	*YAP1*
COCH	*FOXI1*	*KITLG*	*OTOGL*	*SLC26A4*	*ZAK*
COL11A1	*FOXO3A*	*KMT2D*	*P2RX2*	*SLC26A5*	
COL11A2	*FRAS1*	*LARS2*	*PAX3*	*SLC33A1*	
COL2A1	*FREM2*	*LHFPL5*	*PCDH15*	*SLC4A11*	
COL4A3	*GALNS*	*LHX3*	*PDK3*	*SLC4A11*	
COL4A4	*GATA2*	*LHX3*	*PDZD7*	*SLC52A3*	
COL4A5	*GATA3*	*LOR*	*PEX1*	*SLITRK6*	
COL4A6	*GFER*	*LOXHD1*	*PEX10*	*SMPX*	
