# Supplementary material for: Variants encoding a restricted carboxy-terminal domain of SLC12A2 cause hereditary hearing loss in humans
Source: PLoS Genet. 2020 Apr 15;16(4):e1008643. doi: 10.1371/journal.pgen.1008643 (PMC7159186; doi:10.1371/journal.pgen.1008643)
Supplement: S3 Table — (PDF) [file pgen.1008643.s014.pdf]

S3 Table. List of genes categorized in Tier 2 in this study.

Adgrb1	*Ocm*	*Apod*	*Dmd*	*Kcna10*	*Otos*	*Syne4*
Aff3	*Ogfod3*	*Apoe*	*Dnah5*	*Kcne3*	*Otx1*	*Synj2*
Ahrr	*Patl1*	*Aqp4*	*Dnajc5*	*Kcnma1*	*p105*	*Tbx1*
Ahsg	*Pias2*	*Arsa*	*Dnm1*	*Kcnq2*	*p27*	*Tbx15*
Aldh1l1	*Plin4*	*Asic2*	*Duox2*	*Kip1*	*Parp1*	*Tcf4*
Anapc4	*Ppm1a*	*Asic3*	*Dusp6*	*Kit*	*Pax2*	*Tectb*
Ankrd11	*Rims3*	*Atf2*	*Elmod1*	*Kitl*	*Pax8*	*Tfap2a*
Ankrd53	*Rnf10*	*Atoh1*	*Emx2*	*Krt25*	*Pde6b*	*Tgif1*
Ap3s1	*Rsad1*	*Atp8a2*	*Enpp1*	*Krt71*	*Phex*	*Thrb*
Ap4e1	*Scn4a*	*Atp8b1*	*Epha4*	*Lama2*	*Phf20*	*Tmc2*
Ascc2	*Scrib*	*Axin1*	*EPS8*	*Large*	*Phl1*	*TMEM132E*
Atad2b	*Sema3f*	*Barhl1*	*Eps8l2*	*Lhfpl2*	*Phl2*	*Tnfrsf11b*
Atf6b	*Sirt1*	*Bcl2*	*Esr2*	*Lmx1a*	*Pitpnm1*	*Tpo*
B020004J07Rik	*Skida1*	*Bdnf*	*Esrrg*	*Lrig1*	*Pls1*	*Trpa1*
Baiap2l2	*Slc52a2*	*Bloc1s5*	*Etn2*	*Lrig2*	*Pou1f1*	*Tshr*
Bicd1	*Slc7a13*	*Bmp2*	*Fam107b*	*Lrrc4*	*Prkra*	*Ttc26*
Blvrb	*Smo*	*Bmp4*	*Fam20c*	*Lxl2*	*Psap*	*Tub*
Bphl	*Srrm4*	*Bmper*	*Fbxo11*	*Mafb*	*Ptn*	*Tulp1*
Ccdc92	*Taar6*	*Bsn*	*Fbxo2*	*Map1a*	*Rag1*	*Tyrp1*
Ckap4	*Tead1*	*Cacng2*	*Fgf10*	*Map3k1*	*Rb1*	*Ube3b*
Csf3r	*Tmem30b*	*Calb1*	*Fgf20*	*Map3k4*	*Rere*	*Ucn*
Dnajc14	*Tram2*	*Calca*	*Fgfr1*	*Mbp*	*Rgn*	*Vangl1*
Dnase1l2	*Ube2w*	*Car8*	*Fign*	*Mc1r*	*RNR1*	*Vangl2*
Dusp7	*Vti1a*	*Casp3*	*Fos*	*Mcoln3*	*Robo3*	*vglut3*
Emx1	*Wdtc1*	*cdkn1a*	*Foxn1*	*Mcph1*	*Rpl38*	*Wnt5a*
Epn3	*Zfp719*	*cdkn1b*	*Fscn2*	*Mdk*	*Scarb2*	*Wnt7a*
Eps8l1	*Zhx3*	*Cdkn2d*	*Fzd4*	*Mecom*	*Scn8a*	*Zeb1*
Espnl	*Frs2*	*Chrd*	*Gabra5*	*MecP2*	*Sfswap*	*ZNF24*
Exoc4	*Gfpt2*	*Chrna10*	*Gabrb2*	*Mif*	*Sh3pxd2b*	
Fabp1	*Glycam1*	*Chrna9*	*Gabrb3*	*Mkks*	*Slc12a2*	
Fmnl3	*LOC545662*	*Ckmt1*	*Gdf6*	*Mmp14*	*Slc12a6*	
Fnbp1l	*Loxl1*	*Cldn11*	*Gfi1*	*Mpv17*	*Slc12a7*	
Gga1	*Med11*	*Cldn9*	*Gjc3*	*Myc*	*Slc17a5*	
Gm684	*Mocs2*	*Clec16a*	*Gli3*	*Myd88*	*Slc1a3*	
Greb1	*Nos1ap*	*Clic5*	*Golga1*	*Myo15*	*Slc25a21*	
Gtpbp2	*Prdm14*	*Cmah*	*Gpx1*	*Neurod1*	*Slc30a4*	
Hip1r	*PRSS1*	*Col1a1*	*Grid1*	*Nfkb1*	*Slc4a2*	
Hmga1	*Rnf144b*	*COX1*	*Grid2*	*ngfr*	*Slc4a7*	
Hspa5	*Sarnp*	*Cplx1*	*Grm1*	*Nipbl*	*Smn1*	
Htra4	*Thegl*	*Crb1*	*Grxcr2*	*Notch3*	*Smn2*	
Klc2	*Zdhhc11*	*Cs*	*Gsc*	*Npr3*	*Snap25*	
Klhl18	*Abcb1a*	*Csf1*	*Hcn1*	*Nr2f1*	*Sobp*	
Klhl29	*Acan*	*Csf1r*	*Hcn2*	*Ntng2*	*Sod1*	
Lsm1	*Acsl4*	*Ctbp2*	*Hoxa13*	*Ntrk1*	*Sod2*	
Mag	*ADCY1*	*Cyp19a1*	*Hoxb2*	*Ntrk2*	*Sox2*	
Map3k8	*Agap1*	*Cys1*	*Hpn*	*Ntrk3*	*SPATA5*	
Med28	*Agtpbp1*	*DCDC2*	*Irs1*	*Oc90*	*Spns2*	
Nedd4l	*Alg10b*	*Diap3*	*Isl1*	*Oprl1*	*Spry2*	
Nptn	*Ap3b2*	*Dlx1*	*Itpr1*	*OSBPL2*	*Sptbn4*	
Nxn	*Ap3d1*	*Dlx6*	*Kcna1*	*Otor*	*Sun1*	
