# Supplementary material for: Variants encoding a restricted carboxy-terminal domain of SLC12A2 cause hereditary hearing loss in humans
Source: PLoS Genet. 2020 Apr 15;16(4):e1008643. doi: 10.1371/journal.pgen.1008643 (PMC7159186; doi:10.1371/journal.pgen.1008643)
Supplement: S4 Table — (PDF) [file pgen.1008643.s015.pdf]

S4 Table. Primary candidate variants co-segregated with hearing loss in family 1.

Gene symbol	Genome change	Transcript change	Tier	MAF in population database					Zygosity in		
				1000 Genomes	ESP6500	ExAC	HGVD	in house DB	II-1	II-4	III-1
AJAP1	chr1:4772558C>A	NM_018836.3: c.628C>A: p.G210K	3	0	0	0	0	0	None	Hetero	Hetero
NPHP4	chr1:5924546G>A	NM_015102.4: c.3848C>T: p.P1283L	3	0	0	8.29E-06	0	0	None	Hetero	Hetero
SV2A	chr1:149885018GC>G	NM_014849.4: c.374del: p.G125Afs*2	3	0	0	0	0	0	None	Hetero	Hetero
SV2A	chr1:149885020C>A	NM_014849.4: c.373G>T: p.G125C	3	0	0	0	0	0	None	Hetero	Hetero
SLC27A3	chr1:153750718C>T	NM_024330.1: c.1384C>T: p.R462C	3	0	0	0.000124	0	0.000529	None	Hetero	Hetero
PPFIA4	chr1:203022905G>A	NM_001304331.1: c.127T>A: p.S43T	3	0	0	0	0	0.000684	None	Hetero	Hetero
ABI3BP	chr3:100605010A>C	NM_001349329.1: c.640T>G: p.F214V	3	0	0	0	0	0	None	Hetero	Hetero
TMEM108	chr3:133099510C>T	NM_023943.3: c.955C>T: p.R319C	3	0	0	4.12E-05	0	0	None	Hetero	Hetero
TLR2	chr4:154625597C>T	NM_003264.4: c.1538C>T: p.T513M	3	0	0.0003	3.30E-05	0	0	None	Hetero	Hetero
SLC12A2	chr5:127512808G>T	NM_001046.2: c.2941G>T: p.D981Y	2	0	0	0	0	0	None	Hetero	Hetero
CLIC5	chr6:45917008G>T	NM_001114086.1: c.761C>A: p.T254N	1	0	0	0	0	0	None	Hetero	Hetero
PRDM1	chr6:106555348C>T	NM_001198.3: c.2465C>T: p.P822L	3	0	0	1.65E-05	0	0.000511	None	Hetero	Hetero
RAET1E	chr6:150210619C>T	NM_139165.2: c.487G>A: p.E163K	3	0	0	8.24E-06	0	0	None	Hetero	Hetero
ESR1	chr6:152332925G>A	NM_000125.3: c.1231G>A: p.D411N	3	0	0	0	0	0	None	Hetero	Hetero
GNAT3	chr7:80091823C>G	NM_001102386.2: c.715G>C: p.E239Q	3	0	0	0	0	0	None	Hetero	Hetero
MICU3	chr8:16963002A>G	NM_181723.2: c.1166A>G: p.E389G	3	0	0	0	0	0	None	Hetero	Hetero
NOL6	chr9:33463289C>T	NM_022917.4: c.3145G>A: p.V1049M	3	0	0	2.47E-05	0	0	None	Hetero	Hetero
GBA2	chr9:35740010G>C	NM_020944.2: c.1394C>G: p.P465R	3	0	0	0	0	0	None	Hetero	Hetero
DCHS1	chr11:6643315G>A	NM_003737.3: c.9592C>T: p.R3198C	3	0	0	1.65E-05	0	0	None	Hetero	Hetero
USP47	chr11:11955483G>C	NM_017944.3: c.1665+1G>C	3	0	0	0	0	0	None	Hetero	Hetero
DCUN1D5	chr11:102960045T>C	NM_032299.3: c.89A>G: p.Y30C	3	0	0	0	0	0	None	Hetero	Hetero
SPATA19	chr11:133715076C>T	NM_174927.2: c.88G>A: p.V30I	3	0	0	1.65E-05	0	0	None	Hetero	Hetero
LMO3	chr12:16747115del	NM_001243613.1: c.228del: p.L76Ffs*14	3	0	0	0	0	0.000723	None	Hetero	Hetero
YARS2	chr12:32903678G>A	NM_001040436.2: c.1078C>T: p.R360X	3	0	0	2.47E-05	0	0	None	Hetero	Hetero
KRT78	chr12:53233048A>G	NM_173352.3: c.1412T>C: p.I471T	3	0	0	7.41E-05	0	0	None	Hetero	Hetero
TMEM255B	chr13:114504786G>T	NM_182614.3: c.669+1G>T	3	0	0	0	0	0	None	Hetero	Hetero
ADAM20	chr14:70991323C>T	NM_003814.4: c.302G>A: p.R101K	3	0	0	0	0	0	None	Hetero	Hetero
AMN	chr14:103396042C>G	NM_030943.3: c.811C>G: p.R271G	3	0	0	0	0	0	None	Hetero	Hetero
CACNA1H	chr16:1263902A>G	NM_021098.2: c.4900A>G: p.M1634V	3	0	0	0	0	0	None	Hetero	Hetero
TELO2	chr16:1551728G>A	NM_016111.3: c.1426G>A: p.G476S	3	0	7.70E-05	1.65E-05	0	0	None	Hetero	Hetero
NUBP2	chr16:1837789G>A	NM_012225.3: c.446G>A: p.R149H	3	0	0.0002	5.77E-05	0	0	None	Hetero	Hetero
ZNF469	chr16:88495568G>A	NM_001127464.2: c.1690G>A: p.G564R	3	0	0	0	0	0	None	Hetero	Hetero
CANT1	chr17:76993092C>T	NM_138793.3: c.613G>A: p.D205N	3	0	0	3.30E-05	0	0	None	Hetero	Hetero
LDLRAD4	chr18:13645513G>A	NM_181481.4: c.778G>A: p.G260S	3	0	0	0	0	0	None	Hetero	Hetero
IER2	chr19:13264140A>G	NM_004907.2: c.140A>G: p.Y47C	3	0	0	0	0	0	None	Hetero	Hetero
ZNF224	chr19:44611778T>C	NM_013398.3: c.1465T>C: p.C489R	3	0	0	0	0	0	None	Hetero	Hetero
ATRN	chr20:3553455C>T	NM_139321.2: c.1949C>T: p.A650V	3	0	0	8.24E-06	0	0	None	Hetero	Hetero
PDZD4	chrX:153070581G>A	NM_032512.3: c.733C>T: p.R245C	3	0	0	8.24E-05	0	0.000503	None	Hetero	Hetero
