# Supplementary material for: Variants encoding a restricted carboxy-terminal domain of SLC12A2 cause hereditary hearing loss in humans
Source: PLoS Genet. 2020 Apr 15;16(4):e1008643. doi: 10.1371/journal.pgen.1008643 (PMC7159186; doi:10.1371/journal.pgen.1008643)
Supplement: S5 Table — (PDF) [file pgen.1008643.s016.pdf]

S5 Table. Primary candidate variants found in family 2 with hearing loss.

Gene symbo	Genome chagne	Transcript change	Tier	MAF in population database					Zygosity in		
				1000 Genomes	ESP6500	ExAC	HGVD	in house DB	II-3	II-4	III-2
SZT2	chr1:43891188G>A	NM_015284.3: c.2689G>A: p.E897K	3	0	0	0	0	0	None	Hetero	Hetero
SZT2	chr1:43892793G>A	NM_015284.3: c.3205G>A: p.A1069T	3	0	0	0	0.001667	0	Hetero	None	Hetero
SLC12A2	chr5:127512795A>G	NM_001046.2: c.2930-2A>G	2	0	0	0	0	0	None	None	Hetero
C5orf51	chr5:41909849C>T	NM_175921.5: c.209C>T: p.A70V	3	0	0	0	0	0	None	None	Hetero
CASC1	chr12:25261751C>T	NM_018272.4: c.1900G>A: p.E634K	3	0	0	0	0	0	None	None	Hetero
