# Supplementary material for: Variants encoding a restricted carboxy-terminal domain of SLC12A2 cause hereditary hearing loss in humans
Source: PLoS Genet. 2020 Apr 15;16(4):e1008643. doi: 10.1371/journal.pgen.1008643 (PMC7159186; doi:10.1371/journal.pgen.1008643)
Supplement: S6 Table — (PDF) [file pgen.1008643.s017.pdf]

S6 Table. Primary candidate variants found in family 3 with hearing loss.

Gene symbol	Genome change	Transcript change	Tier	MAF in population database					Zygosity in		
				1000 Genomes	ESP6500	ExAC	HGVD	in house DB	II-3	II-4	III-2
SLC12A2	chr5:127512829C>A	NM_001046: c.2962C>A: p.P988T	2	0	0	0	0	0	None	None	Hetero
TECTA	chr11:121028739G>C	NM_005422: c.4495G>C: p.D1499H	1	0	0	0	0.001166	0.001166	Hetero	None	Hetero
ACAN	chr15:89381906C>G	NM_013227.3: c.83C>G: p.S28W	2	0.0002	0	2.71E-05	0.003709	0.003709	None	Hetero	Hetero
