# Supplementary material for: Variants encoding a restricted carboxy-terminal domain of SLC12A2 cause hereditary hearing loss in humans
Source: PLoS Genet. 2020 Apr 15;16(4):e1008643. doi: 10.1371/journal.pgen.1008643 (PMC7159186; doi:10.1371/journal.pgen.1008643)
Supplement: S7 Table — (PDF) [file pgen.1008643.s018.pdf]

**S7 Table.** List of deafness genes for targeted sequencing.

| | | | |
|---|---|---|---|
| *ACTG1* | *ESPN* | *OTOF* | *MTTE* |
| *ADCY1* | *ESRRB* | *OTOG* | *MTTK* |
| *ADGRV1* | *EYA1* | *OTOGL* | *MTTL1* |
| *AIFM1* | *EYA4* | *P2RX2* | *MTTS1* |
| *ALMS1* | *FAM65B* | *PAX3* | *MTRNR1* (rs3888511) |
| *ATP2B2* | *FOXI1* | *PCDH15* | *MTRNR1* (rs267606617) |
| *ATP6V0A4* | *GATA3* | *PDZD7* | *MTRNR1* (rs267606619) |
| *ATP6V1B1* | *GIPC3* | *PNPT1* | |
| *BDP1* | *GJB2* | *POU3F4* | |
| *BSND* | *GJB3* | *POU4F3* | |
| *CABP2* | *GJB6* | *PRPS1* | |
| *CACNA1D* | *GPSM2* | *PTPN11* | |
| *CATSPER2* | *GRHL2* | *PTPRQ* | |
| *CCDC50* | *GRXCR1* | *RDX* | |
| *CD164* | *GRXCR2* | *S1PR2* | |
| *CDC14A* | *HARS2* | *SEMA3E* | |
| *CDH23* | *HGF* | *SERPINB6* | |
| *CEACAM16* | *HOMER2* | *SIX1* | |
| *CHD7* | *HOXA1* | *SIX5* | |
| *CIB2* | *HSD17B4* | *SLC12A2* | |
| *CLCNKA* | *ILDR1* | *SLC17A8* | |
| *CLCNKB* | *KARS* | *SLC22A4* | |
| *CLDN14* | *KCNE1* | *SLC26A4* | |
| *CLIC5* | *KCNJ10* | *SLC26A5* | |
| *CLPP* | *KCNQ1* | *SLITRK6* | |
| *CLRN1* | *KCNQ4* | *SMPX* | |
| *COCH* | *KITLG* | *SNAI2* | |
| *COL11A1* | *LARS2* | *SOX10* | |
| *COL11A2* | *LHFPL5* | *STRC* | |
| *COL2A1* | *LOXHD1* | *SYNE4* | |
| *COL4A3* | *LRTOMT* | *TBC1D24* | |
| *COL4A4* | *MARVELD2* | *TECTA* | |
| *COL4A5* | *MCM2* | *TIMM8A* | |
| *COL4A6* | *MET* | *TJP2* | |
| *COL9A1* | *MIR96* | *TMC1* | |
| *COL9A2* | *MITF* | *TMEM126A* | |
| *CRYM* | *MSRB3* | *TMEM132E* | |
| *DCDC2* | *MYH14* | *TMIE* | |
| *DFNA5* | *MYH9* | *TMPRSS3* | |
| *DFNB59* | *MYO15A* | *TNC* | |
| *DIABLO* | *MYO3A* | *TPRN* | |
| *DIAPH1* | *MYO6* | *TRIOBP* | |
| *DIAPH3* | *MYO7A* | *TSPEAR* | |
| *DLX5* | *NARS2* | *TWNK* | |
| *DSPP* | *NDP* | *USH1C* | |
| *EDN3* | *NF2* | *USH1G* | |
| *EDNRB* | *NOG* | *USH2A* | |
| *ELMOD3* | *OPA1* | *WFS1* | |
| *EPS8* | *OSBPL2* | *WHRN* | |
| *EPS8L2* | *OTOA* | | |
