# Supplementary material for: Variants encoding a restricted carboxy-terminal domain of SLC12A2 cause hereditary hearing loss in humans
Source: PLoS Genet. 2020 Apr 15;16(4):e1008643. doi: 10.1371/journal.pgen.1008643 (PMC7159186; doi:10.1371/journal.pgen.1008643)
Supplement: S8 Table — (PDF) [file pgen.1008643.s019.pdf]

S8 Table. Primers used in this study.

Target gene and locus	Purpose	Forward primer (5'-3')	Reverse primer (5'-3')
H. sapiens SLC12A2 exon 21	Validation by Sanger sequencing	gtaaaacgacggccagtGCCACAGTTCAACATCTTCTCAAC	caggaaacagctatgacACCGGCTGTCTGGGTCTAATA
H. sapiens TECTA exon 13	Validation by Sanger sequencing	TGCTGCCTCCTACAAATTCC	CTAGCCAACCCCTTGTTTCC
H. sapiens ACAN exon 3	Validation by Sanger sequencing	gtaaaacgacggccagtTGCATTGCTGGAAGGATGGA	caggaaacagctatgaccCCAGCTCCATGTCACAGAGG
H. sapiens D1S80 (MCT118)	Paternity test	GTCTTGTTGGAGATGCACGTGCCCCTTGC	GAAACTGGCCTCCAAACACTGCCCGCCG
H. sapiens D17S5 (YNZ22)	Paternity test	AAACTGCAGAGAGAAAGGTCGAAGAGTGAAGTG	AAAGGATCCCCCACATCCGCTCCCCAAGTT
GRCh37.chr5:127511985–127514491	Construction of minigene	aaaaagcaggctACTCAGCTGTTTTAACTCCTGGGAA	agaaagctgggtATGCTGGCAAAATTTGAATGGGGTC
Minigene reporter transcript	RT-PCR	gtaaaacgacggccagtAGCACCTTTGTGGTTCTCACT	caggaaacagctatgaccGTGCAGCACTGATCCACGAT
H. sapiens and *M. fascicularis SLC12A2,* exon 21–included	qRT-PCR	ACACACAAAGTTGAGGAAGAGGA	GGCACAATAGGGCCTTTGGA
H. sapiens and *M. fascicularis SLC12A2,* exon 21–skipped	qRT-PCR	CTGGCACCAAGGATGTGGTA	GGGCCTTTGGATTCTTTGTGT
H. sapiens and *M. fascicularis GAPDH*	qRT-PCR	CACCATCTTCCAGGAGCGAG	GACTCCACGACGTACTCAGC
M. musculus Slc12a2, exon 21–included	qRT-PCR	CAGAAAGATGAGGAAGAGGATGGC	GCACAATAGGGCCTTTGGATTC
M. musculus Slc12a2, exon 21–skipped	qRT-PCR	CCTGGTACCAAGGATGTGGT	AGGGCCTTTGGATTCTTTCTGT
M. musculus Gapdh	qRT-PCR	AAATGGTGAAGGTCGGTGTG	TGAAGGGGTCGTTGATGG
H. sapiens and *M. fascicularis SLC12A2*	RT-PCR	GGCACCAAGGATGTGGTAGT	CATCGCTCTCCGGTCATGG
M. musculus Slc12a2	RT-PCR	ACCTGGTACCAAGGATGTGG	TTCTGAAACTGTGTGCTAGC
H. sapiens GAPDH	RT-PCR	CCATGGAGAAGGCTGGGG	CAAAGTFGTCATGGATGACC
M. fascicularis GAPDH	RT-PCR	GCCGCATTTTCTCTTGCATCGCC	GCCCCAGCCTTCTCCGTGGT
M. musculus Gapdh	RT-PCR	AACGGGAAGCCCATCACC	CAGCCTTGGCAGCACCAG

Nucleotides in lower case indicate adapters or sequencing primers.
